# Genome-Wide Sequence Variation Identification and Floral-Associated Trait Comparisons Based on the Re-sequencing of the ‘Nagafu No. 2’ and ‘Qinguan’ Varieties of Apple (*Malus domestica* Borkh.)

**DOI:** 10.3389/fpls.2016.00908

**Published:** 2016-06-27

**Authors:** Libo Xing, Dong Zhang, Xiaomin Song, Kai Weng, Yawen Shen, Youmei Li, Caiping Zhao, Juanjuan Ma, Na An, Mingyu Han

**Affiliations:** College of Horticulture, Northwest Agriculture and Forestry UniversityYangling, China

**Keywords:** genome variation, INDELs, re-sequencing, flowering genes, apple (*Malus domestica* Borkh.)

## Abstract

Apple (*Malus domestica* Borkh.) is a commercially important fruit worldwide. Detailed information on genomic DNA polymorphisms, which are important for understanding phenotypic traits, is lacking for the apple. We re-sequenced two elite apple varieties, ‘Nagafu No. 2’ and ‘Qinguan,’ which have different characteristics. We identified many genomic variations, including 2,771,129 single nucleotide polymorphisms (SNPs), 82,663 structural variations (SVs), and 1,572,803 insertion/deletions (INDELs) in ‘Nagafu No. 2’ and 2,262,888 SNPs, 63,764 SVs, and 1,294,060 INDELs in ‘Qinguan.’ The ‘SNP,’ ‘INDEL,’ and ‘SV’ distributions were non-random, with variation-rich or -poor regions throughout the genomes. In ‘Nagafu No. 2’ and ‘Qinguan’ there were 171,520 and 147,090 non-synonymous SNPs spanning 23,111 and 21,400 genes, respectively; 3,963 and 3,196 SVs in 3,431 and 2,815 genes, respectively; and 1,834 and 1,451 INDELs in 1,681 and 1,345 genes, respectively. Genetic linkage maps of 190 flowering genes associated with multiple flowering pathways in ‘Nagafu No. 2,’ ‘Qinguan,’ and ‘Golden Delicious,’ identified complex regulatory mechanisms involved in floral induction, flower bud formation, and flowering characteristics, which might reflect the genetic variation of the flowering genes. Expression profiling of key flowering genes in buds and leaves suggested that the photoperiod and autonomous flowering pathways are major contributors to the different floral-associated traits between ‘Nagafu No. 2’ and ‘Qinguan.’ The genome variation data provided a foundation for the further exploration of apple diversity and gene–phenotype relationships, and for future research on molecular breeding to improve apple and related species.

## Introduction

The domesticated apple (*Malus domestica* Borkh.) is one of the most commercially important fruit worldwide, with over 60 million tons produced each year (Food and Agriculture Organization of the United Nations, 2013^1^). There are more than 10,000 documented cultivars of apples (Hummer and Janick, 2009), resulting in a range of desired characteristics. China is the leading apple-producing country, with a planting area of 3.1 million hectares and production of 33 million tons annually^1^. ‘Nagafu No. 2,’ which accounts for more than 65% of the total cultivated area, is the dominant cultivar in China. However, ‘Nagafu No. 2’ apples have difficulty forming flower buds and have an alternate bearing problem, which results in unstable and low fruit production. ‘Qinguan’ is an elite variety bred in China with strong disease resistance, easy flowering, high yield, and easy management. ‘Nagafu No. 2’ and ‘Qinguan’ are important materials for apple breeding and genetic research in China because of their different flowering and drought resistance characteristics. However, the genetic basis underlying these differences and the associated genomic information for these two varieties are poorly understood.

Genomic sequences of perennial fruit crops, such as grape (Jaillon et al., 2007), apple (Velasco et al., 2010), peach (Verde et al., 2013), pear (Wu et al., 2013), and sweet orange (Xu et al., 2013), have been determined over the past 10 years. The first physical map of the apple genome was constructed from bacterial artificial chromosome clones by (Han et al., 2007) and covered ∼927 Mb. Numerous expressed sequence tags (ESTs) were collected in apple from libraries covering a variety of genotypes and tissues, under different experimental conditions (Gasic et al., 2009)^2^, and have allowed the efficient development of DNA-based markers (Park et al., 2006), gene discovery (Chagné et al., 2008), and comparative genomics (Gasic et al., 2009). However, compared with other model plants, the study of the apple genome is still in its infancy.

Next-generation sequencing (NGS) technologies have enabled the identification of genome-wide patterns of genetic variation in perennial fruit crops in a rapid, efficient, relatively low cost, and high-throughput manner (Chagné et al., 2012; Montanari et al., 2013; Cao et al., 2014). Genetic variation comprises structural alterations and sequence variations. Sequence variations are categorized into single nucleotide polymorphisms (SNPs), short sequence insertions and deletions (INDELs), microsatellites (simple sequence repeats), and transposable elements (Zheng et al., 2011). To date, whole-genome INDELs and SNPs have been developed for evolutionary and functional studies in many plants, including apple (Velasco et al., 2010), pear (Montanari et al., 2013), and peach (Cao et al., 2014). NGS was used to detect SNPs covering the apple genome and the Illumina InfiniumH II system was developed as a medium- to high-throughput SNP screening tool to identify allelic variation in apple (Chagné et al., 2012). Additionally, NGS was used to detect SNPs in the pear genome and a medium-throughput SNP assay was designed (Montanari et al., 2013). Incorporation of the new pear SNPs into the apple 8 K array enabled the study of SNP transferability not only within the genus *Pyrus*, but also between the genera *Malus* and *Pyrus*. In addition, 10 wild and 74 cultivated peach varieties were resequenced on a large scale and 4.6 million SNPs were identified (Cao et al., 2014). Structural alterations are generally described as copy number variations and presence/absence variations, which include large-scale duplications, insertions, deletions, translocations, and inversions (Zheng et al., 2011). Multiple repeats of a promoter segment cause transcription factor autoregulation in red apples (Espley et al., 2009).

Molecular mechanisms regulating the flowering process have been extensively studied. Four major flowering promotion pathways (photoperiodic, autonomous, vernalization-response, and gibberellin) have been established in model annual plants (Andres and Coupland, 2012). In perennials, however, the molecular mechanisms controlling flowering are poorly understood. In apple, many of the homeotic genes of floral development have been isolated and their expression patterns examined (Koutinas and Pepelyankov, 2010). Recently, some genes have been functionally characterized, for example, the overexpression of an FT-homologous gene in apple induced early flowering in annual and perennial plants (Traenkner et al., 2010). *MdFT1* and/or *MdFT2* might also be associated with flowering and fruiting by interacting with proteins of the *TCP* and *VOZ* families of transcription factors in apple (Mimida et al., 2011). To date, however, there have been no studies on the genetic control of floral initiation and development in apple using high-throughput re-sequencing technology.

We used Solexa sequencing technology and the Illumina HiSeq^TM^ 2000 to re-sequence the genomes of ‘Nagafu No. 2’ and ‘Qinguan,’ and to analyze their genetic structures. We developed effective DNA markers to explore agronomic traits-related genes by comparing the ‘Nagafu No. 2’ and ‘Qinguan’ sequences with the published reference genome. A comparison of the variation data defined potential genomic regions and metabolic pathways related to floral-associated traits. The genomic resources provided here are useful for comparative genomics and molecular breeding in apple and related species.

## Materials and Methods

### Plant Material and Sample Collection

The materials used in this study were collected from the Apple Demonstration Nursery of Yangling Modern Agriculture Technology Park (Northwest Agriculture and Forestry University) in the Shaanxi Province of China (34°52′ N, 108°7′ E). Young leaves of apple varieties (*Malus domestica* Borkh.) ‘Nagafu No. 2’ and ‘Qinguan’ were used as materials for re-sequencing. Physiological differentiation stage of apple flower bud was from May 5th, 2014 (ES, early stage) to June 25th, 2014 (LS, late stage). And bud growth including length, width, and fresh weight during the flower bud physiological stage (ES, MS, and LS) can be seen in our published paper (Xing et al., 2015). Leaf and bud samples of ‘Nagafu No. 2’ and ‘Qinguan’ were collected for gene expression analysis on June 5th, 2014, 45 days after full bloom (MS, the middle stage of flower bud physiological differentiation). Each reaction was performed with three replicates.

### DNA Sequencing and Mapping

Genomic DNA was extracted from young leaves of ‘Nagafu No. 2’ and ‘Qinguan’ using a modified CTAB method (Zhang et al., 2011). The DNA was then randomly sheared and then purified using a QIAquick PCR Purification Kit 28104 (Qiagen, Beijing, China). Adaptor ligation and DNA cluster preparation were performed, followed by Solexa sequencing using an Illumina HiSeq^TM^ 2000. Low-quality reads (<20), reads with adaptor sequences, and duplicated reads were eliminated. The remaining high-quality data were used for mapping.

We used the published genome of ‘Golden Delicious’ as a reference (Velasco et al., 2010). We mapped the reads of each accession to the scaffold of the reference genome using BWA software^3^ under default parameters, with a small modification: allowing no more than three mismatches in the sequence and not allowing gaps (-o 0). Reads that aligned to more than one position of the reference genome were filtered and used to determine reads mapping to multiple positions in the reference and unmapped reads. After mapping, the reads were sorted by their scaffold coordinates. The mapping result was used to detect DNA polymorphisms, such as SNPs, INDELs, and SVs.

### Detection of SNP, INDEL, and SV Polymorphisms

SAMtools software^4^ was used to detect SNPs using the following parameters: u -fa -C 50 – bcftools view -I -N- b-v -c -g. The detected SNPs were screened using the following criteria: coverage depth ≥2×, heterozygous locus ≥3×, average depth ≤3×, no less than 20 for the quality value of the genomic type, and discarding SNPs detected in repeat regions.

Structural variations (SVs) were detected using Pindel^5^ and Breakdancer^6^ software, with their default parameters. To obtain reliable SVs, the detected SVs were returned to the pair-end reads alignments between samples and the reference, and were validated under the following criteria: 2× to 100× for coverage depth and more than 20 for SV quality. We detected insertion (INS), deletion (DEL), deletion including insertion (IDE), inversion (INV), intra-chromosomal translocation (ITX), and inter-chromosomal translocation (CTX) SVs. INDELs were defined as the insertion or deletion of 1–5 bp.

### Annotation of SNPs, INDELs, and SVs

The locations of SNPs, INDELs, and SVs were based on the annotation of gene models from the reference genome database^7^. Polymorphisms in the gene region and other genome regions were annotated as genic and intergenic, respectively. The genic SNPs and SVs were classified as exonic and intronic based on their location. SNPs in coding DNA sequences (CDSs) were further separated into synonymous and non-synonymous using Genewise^8^.

### Identifying Genes Associated with DNA Polymorphisms and Their Functional Analysis

Using the ‘Golden Delicious’ gene set as the reference, genes containing DNA polymorphism were identified in the ‘Nagafu No. 2’ and ‘Qinguan’ apple varieties. The genes associated with the DNA polymorphisms were annotated in databases, such as Swissprot, COG, Nr, and GO, for both ‘Nagafu No. 2’ and ‘Qinguan’ apples (Ashburner et al., 2000; Tatusov et al., 2000; Apweiler et al., 2004). In addition, the KEGG^9^ analysis of genes with DNA polymorphisms identified those enriched in particular pathways, based on the hypergeometric distribution test (Kanehisa et al., 2004). The Fisher’s exact test was used to identify pathways significantly enriched (*P*-value <0.1) with related genes.

### Cloning of the *FT* Promoter Region

The primers used to amplify the *FT* promoter region were based on those described in a previous study on apples (Traenkner et al., 2010). Using the promoter sequence obtained for *FT*, primers were redesigned to compare sequences among different cultivars. PCR products were analyzed on 1.0% agarose gels, and for each reaction product, a single fragment was recovered from gels and purified using a DNA purification kit (Takara, Ohtsu, Japan). The fragment was then ligated into the plasmid pMD18-T vector, transformed into *Escherichia coli* DH5α competent cells (Takara, Ohtsu, Japan), and sequenced (Sangon Biotech, Shanghai, China). In addition, we predicted the transcription factor binding sites in the *FT* promoters of the two apple varieties using Plant CARE^10^.

### RNA Extraction, cDNA Synthesis, and qRT-PCR Validation of Flowering Genes

Total RNA was isolated from each sample using a modified CTAB method (Xing et al., 2014). The concentration of total RNA was measured using a Nanodrop 2000 after DNase I digestion of genomic DNA. First-strand cDNA was synthesized from 4 μg of DNA-free RNA using a Revert Aid^TM^ First-Strand cDNA Synthesis Kit (Fermentas, Glen Burnie, MD, USA). The cDNA was diluted 10-fold, and 2 μL was used as the template for qRT-PCR analyses. Each qRT-PCR reaction mixture contained 10.0 μL SYBR Premix Ex Taq^TM^ (Takara, Ohtsu, Japan), 0.4 μL each primer (10 μM), 2 μL cDNA, and 7.2 μL RNase-free water in a total volume of 20 μL. The reactions were incubated in an iCycler iQ5 (BIO-RAD) for 30 s at 95°C; followed by 40 cycles of 5 s at 95°C and 35 s at 60°C. Then, an additional 81 cycles were run for the melt curve. The qRT-PCR primers were designed using primer 3 software^11^. The primers used in the qRT-PCR experiments are listed in **Supplementary Table S1**. Each reaction was performed with three replicates.

### Statistical Analysis

The expression levels of the flowering genes in the buds and leaves of ‘Nagafu No. 2’ and ‘Qinguan’ apple varieties were analyzed by one-way analyses of variance with Tukey–Kramer multiple comparison tests using DPS software, version 7.0 (Zhejiang University, Hangzhou, China).

## Results

### Agronomic and Floral-Associated Traits of the Apple Varieties Used for Re-sequencing

‘Nagafu No. 2’ is a hybrid progeny of ‘Red Delicious’ and ‘Ralls Genet’ introduced to China in 1966. It has the good agronomic traits of fleshy fruit, delicate flavor, and great storability; however, it has difficulty flowering (The flowering rate in 6-year-old trees is just ∼20%), poor disease resistance, and environmental adaptation (**Table 1**). ‘Qinguan’ is a hybrid progeny of ‘Golden Delicious’ and ‘Cockscomb’ that shows high drought resistance during the whole growth period. ‘Qinguan’ flowers easily (The flowering rate in 6-year-old trees is ∼70%), has a high yield and is easy to manage (**Table 1**). These apple varieties differ in a number of floral-associated traits (**Table 1**). The two easy-flowering varieties (‘Qinguan’ and ‘Golden Delicious’) had higher proportions of spurs, budding rates, flower formation rates, and formed spurs and full blooms at earlier dates compared with ‘Nagafu No. 2’ (**Table 1**). The variation in the biological traits of these three varieties provides the basis to study gene-trait associations by examining the sequence polymorphisms and structural variations at the whole-genome level.

**Table 1:** Agronomic and floral-associated traits of the apple varieties used for re-sequencing.

	Varieties		
	
Floral trait	‘Nagafu No.2’	‘Qinguan’	‘Golden delicious’
Proportion of spur	19.5 ± 2.1% c	44.3 ± 2.5% a	37.9 ± 2.2% b
Budding rate	61.8 ± 2.7% b	66.7 ± 3.5 % a	64.3 ± 3.4% ab
Flower formation rate	19.3 ± 1.9% c	72.0 ± 2.6% a	65.3 ± 3.2% b
Date of forming spur	15th–20th May	8th–12th May	10th–14th May
Full-bloom stage	15th–21st April	10th–16th April	12th–16th April


*The data are shown as means ± standard errors and were collected from plants grown in an experimental field in Yangling over three consecutive years (2011–2013). Different lowercase letters in the same row indicate significant differences at *P* < 0.05.*

### Mapping of Re-sequencing Reads to the Reference Apple Genome

Whole-genome sequencing produced 171,118,509 reads (91,406,980 reads for ‘Nagafu No. 2’ and 79,711,529 reads for ‘Qinguan’; **Table 2**). For the clean reads, 80.22% from ‘Nagafu No. 2’ and 81.52% from ‘Qinguan’ were mapped successfully onto the reference genome of ‘Golden Delicious’ (**Table 2**). Details of the data quality, including base distribution, cycle average phred score, and quality distribution, of the two apple varieties are presented in **Supplementary Figures S1** and **S2**, respectively. The sequencing depth distribution and the distribution on chromosomes are shown in **Supplementary Figures S3** and **S4**, respectively. Compared with the reference genome, the identities of ‘Nagafu No. 2’ and ‘Qinguan’ were 98.62% and 98.7%, respectively (**Table 2**). The re-sequencing data have been deposited in NCBI Sequence Read Archive (SRA^12^). And accession number was SRP072330.

**Table 2:** Summary of the original sequencing data of ‘Nagafu No. 2’ and ‘Qinguan.’

	Index								
	
Variety	Total reads	PE (%)	SE (%)	Total bases	GC (%)	Q30 (%)	Mapped (%)	Identity (%)	Depth	Coverage (%)
‘Nagafu No.2’	91,406,980	54.8	25.41	18,464,209,960	40.20	83.21	80.22	98.62	31.07	96.94
‘Qinguan’	79,711,529	56.61	24.91	16,101,728,858	40.14	82.82	81.51	98.7	27.78	97.36


*PE, pair-end reads; SE, single-end reads; Q30 (%) indicates the percentage of sequences with an error rate lower than 1%.*

### Detection and Characteristics of SNPs, SVs, and INDELs

Based on the alignment results between samples and the reference genome, 2,771,129 genome-wide SNPs (**Supplementary Table S2**), 82,663 SVs (**Supplementary Table S2** and Additional file 1), and 1,572,803 INDELs were detected in ‘Nagafu No. 2,’ while 2,262,888 genome-wide SNPs, 63,764 SVs and 1,294,060 INDELs were detected in ‘Qinguan’ (**Supplementary Table S2**). The average densities of detected SNPs, SVs, and INDELs for ‘Nagafu No. 2’ and ‘Qinguan,’ when compared to samples the reference genome, were 4,546.9 and 3,746.0 SNPs per Mb, respectively, 135.9 and 105.3 SVs per Mb, respectively, and 2,584.1 and 2,145.4 INDELs per Mb, respectively (**Supplementary Table S2**).

### Distribution of SNPs, SVs, and INDELs

**FIGURE 1:**
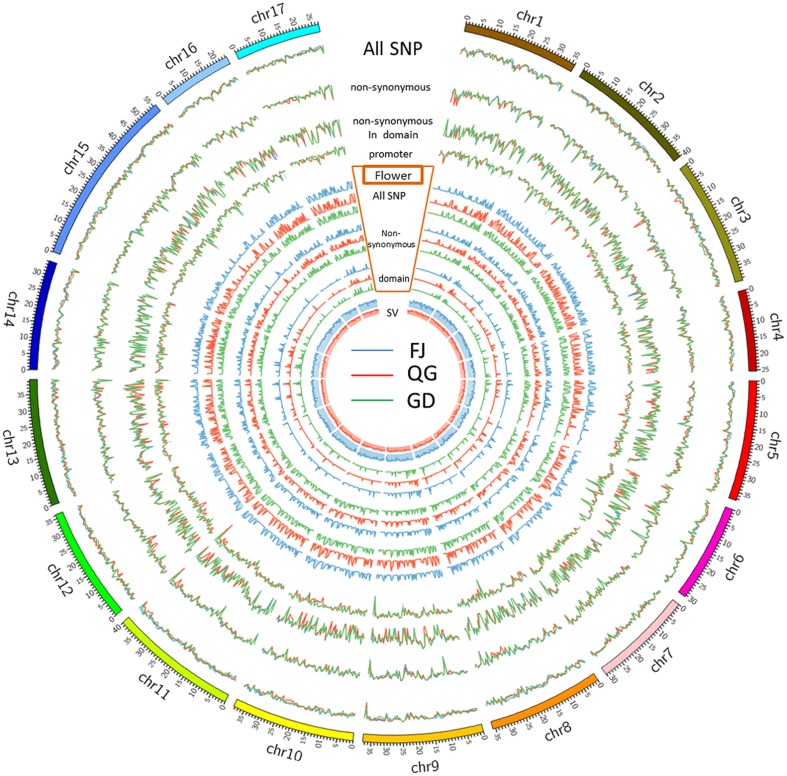
**Genetic variations and distributions of DNA polymorphisms (SNPs and SVs) and flowering genes in samples compared with the ‘Golden Delicious’ genome**.

Detailed distribution information for the SNPs, SVs, and INDELs in both ‘Nagafu No. 2’ and ‘Qinguan’ samples identified the landscape of genetic variation in the two apple varieties (**Figure 1**, **Supplementary Table S2** and Additional file 2). Among the variations detected between the samples and reference genome ‘Golden Delicious,’ the highest numbers of SNPs, 231,577 in ‘Nagafu No. 2,’ and 193,484 in ‘Qinguan,’ were observed on chromosome 15, while the lowest numbers of SNPs, 104,010 in ‘Nagafu No. 2’ and 99,017 in ‘Qinguan,’ were observed on chromosome 16 (**Supplementary Table S2**). The highest numbers of SVs were observed on chromosome 15, 6,817 in ‘Nagafu No. 2’ and 5,475 in ‘Qinguan,’ while the lowest numbers of SVs, 3,448 in ‘Nagafu No. 2’ and 2,786 in ‘Qinguan’ were observed on chromosome 16 (**Supplementary Table S2**). The highest numbers of INDELs, 131,976 in ‘Nagafu No. 2’ and 109,667 in ‘Qinguan,’ were observed on chromosome 15, and the lowest numbers of INDELs, 60,055 in ‘Nagafu No. 2’ and 56,509 in ‘Qinguan,’ were observed on chromosome 16 (**Supplementary Table S2**).

Our result showed that the distribution of polymorphisms was uneven within chromosomes (**Figure 1**). The three peripheral circles represent the distribution of polymorphisms in ‘Nagafu No. 2,’ ‘Qinguan,’ and ‘Golden Delicious,’ respectively (**Figure 1**). These circles indicate the statistical information for total SNPs (blue line), non-synonymous SNPs (red line), and SNPs in structural domains (green line) on each chromosome. The promoter circles indicate the SNP numbers of ‘Nagafu No. 2’ (blue line), ‘Qinguan’ (red line), and Golden Delicious (green line), which are located in the 200 bp upstream of each coding region (**Figure 1**). Inside the promoter circles, there are three grouped circles, which represent the distribution of flowering genes on each chromosome of ‘Nagafu No. 2,’ ‘Qinguan,’ and ‘Golden Delicious.’ We chose flowering genes mainly based on previous research in the model plant *Arabidopsis thaliana* (Blümel et al., 2015). The blue line indicates the total SNPs related to flowering, the red line indicates the sense mutation of SNPs related to flowering, and the green line indicates the SNPs related to flowering in structural domains (**Figure 1**). The inner circles represent the SV distribution on each chromosome of ‘Nagafu No. 2’ (blue line) and ‘Qinguan’ (red line).

In addition, detailed information on the distribution of SNPs and SVs detected between ‘Nagafu No. 2’ and ‘Qinguan’ on the 17 chromosomes is shown in **Supplementary Figure S5**. The distribution of polymorphisms was uneven within chromosomes, as well as between each chromosome of ‘Nagafu No. 2’ and ‘Qinguan’ (**Supplementary Figure S5**), and all of the chromosomes in both varieties were comprised of a mixture of SNP-dense and -sparse regions (**Supplementary Figure S5**). The number of SNPs (133,130) and SVs (4,016) in ‘Nagafu No. 2’ were significantly higher than in ‘Qinguan’ (115,431 SNPs and 3,245 SVs) for chromosome 1. Other chromosomes showed similar results (**Supplementary Figure S5**). Additionally, the distribution patterns of the two polymorphism types were similar in the two varieties (**Supplementary Figure S5**).

### Characteristics of SNPs, SVs, and INDELs

The SNPs detected when comparing the two samples with the reference genome were classified as transitions (C/T and G/A) or transversions (C/G, T/A, A/C, and G/T) based on nucleotide substitutions (**Supplementary Table S3**). The proportions of transitions and transversions on individual chromosomes were very similar, but the proportions of transitions were significantly higher than the proportions of transversions in both samples (**Supplementary Table S3**). The proportions of heterozygosity were significantly higher than the proportions of homozygosity in the two samples, and the proportion of heterozygosity in ‘Qinguan’ was significantly higher than the proportion of heterozygosity in ‘Nagafu No. 2’ (**Supplementary Table S3**). The highest proportions of SNP heterozygosity, 89.66 and 93.67%, were observed on chromosome 7 of ‘Nagafu No. 2’ and ‘Qinguan,’ respectively, while the lowest proportions of SNP heterozygosity, 4.09 and 86.08%, were observed on chromosome 2 of ‘Nagafu No. 2’ and chromosome 8 of ‘Qinguan,’ respectively (**Supplementary Table S3**).

**FIGURE 2:**
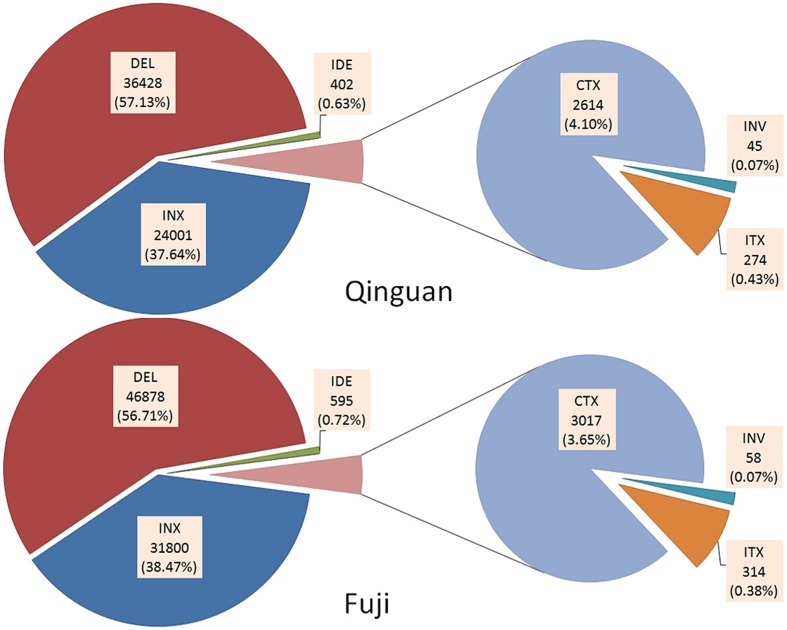
**Annotation of SVs identified in ‘Nagafu No. 2’ and ‘Qinguan’ apple varieties.** Structural variations (SVs) were classified as INS (Insertion), DEL (Deletion), IDE (Deletion including insertion), INV (Inversion), ITX (Intra-chromosomal Translocation), or CTX (Inter-chromosomal Translocation) based on the annotation of the ‘Golden Delicious’ genome.

In addition, six types of SVs, INS, DEL, IDE, INV, ITX, and CTX, were identified in both cultivars, based on the annotation of ‘Golden Delicious’ genome (**Supplementary Table S4**). In ‘Nagafu No. 2’ and ‘Qinguan,’ 63,764 and 82,663 SVs, respectively, were detected (**Supplementary Table S2**). There was no significant difference in the percentage of each SV between ‘Nagafu No. 2’ and ‘Qinguan.’ Among them, DEL and INS were the major SVs, with 46,878 (56.71%) and 31,800 (38.47%), respectively, for ‘Nagafu No. 2,’ and 36,428 (57.13%) and 24,001 (37.64%), respectively, for ‘Qinguan’ (**Figure 2**). INX [314 (0.38%), and 274 (0.43%) for ‘Nagafu No. 2 and ‘Qinguan,’ respectively] and INV [314 (0.07%) and 45 (0.07%) for ‘Nagafu No. 2 and ‘Qinguan,’ respectively] were the minor SVs in the two varieties (**Figure 2** and **Supplementary Table S4**). Among the variations detected between samples and ‘Golden Delicious,’ the highest densities of SVs were 162.97 per Mb, observed on chromosome 11 of ‘Nagafu No. 2’ and 114.42 per Mb on chromosome 17 of ‘Qinguan.’ The lowest densities of SVs were 111.31 per Mb and 89.94 per Mb observed on chromosome 1 in ‘Nagafu No. 2’ and ‘Qinguan,’ respectively (**Supplementary Table S4**).

### Annotation of Genes with Genome Variations and Their Function Analysis

**FIGURE 3:**
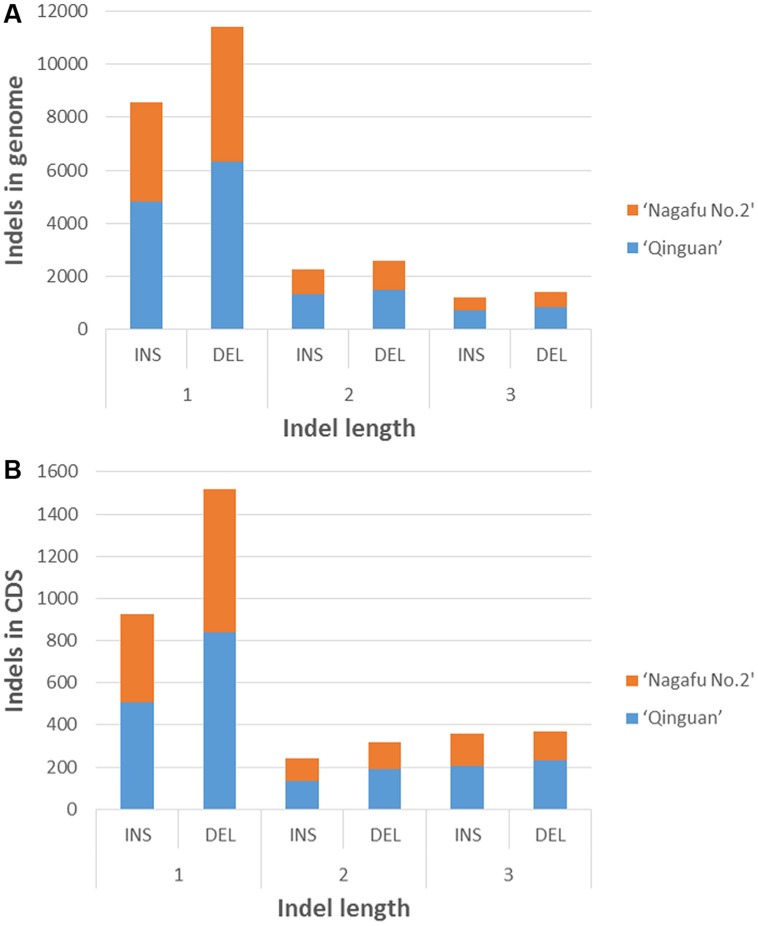
**Distribution of 1- to 3-bp INDELs in ‘Nagafu No. 2’ and ‘Qinguan’ apple varieties.** **(A)** Number of INDELs of different lengtha in the genomic regions; **(B)** Number of INDELs of different lengths in the CDS regions.

**FIGURE 4:**
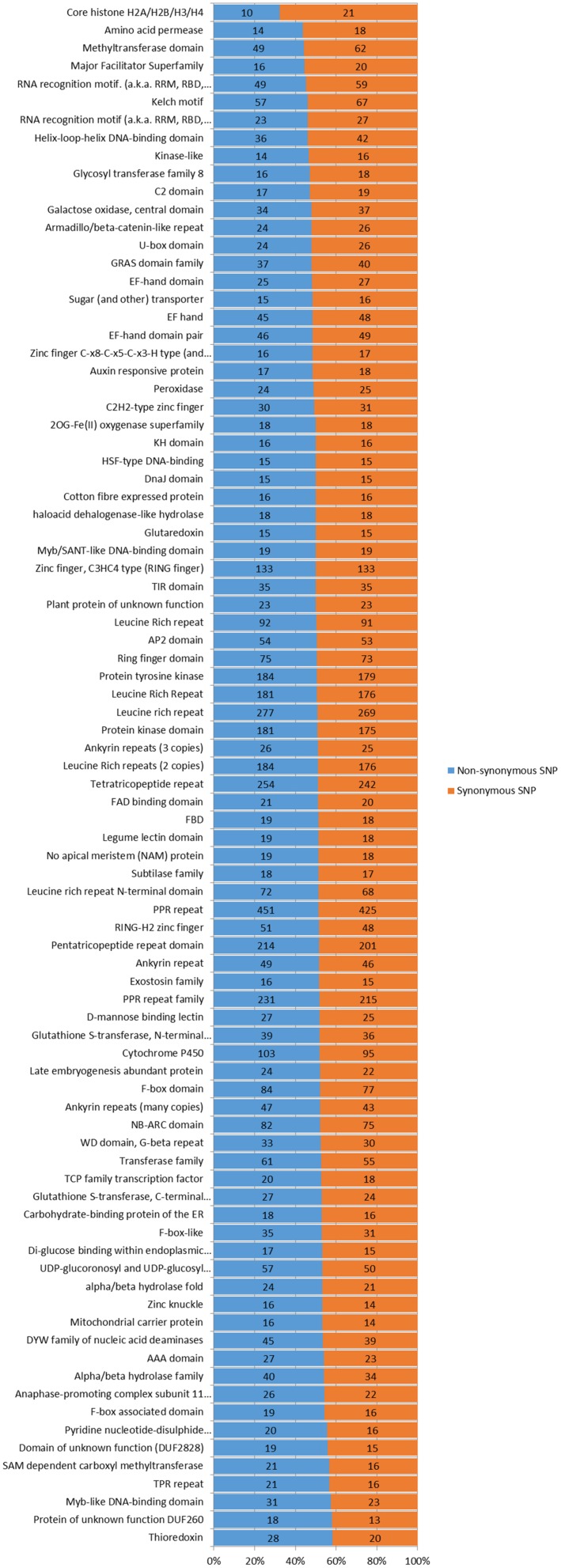
**Number and distribution of non-synonymous and synonymous SNPs in different Pfam genes in the apple genomes.** Pfam gene families with 30 or more non-synonymous and synonymous SNPs were analyzed and listed. The Pfam genes are arranged based on the percentages of non-synonymous and synonymous SNP sites. The chi-square significance of the observed non-synonymous and synonymous SNP distributions for each Pfam group is shown: *P*-value <0.001.

In the genomic regions of ‘Nagafu No. 2’ and ‘Qinguan,’ 4,827 and 3,719 INS, respectively, and 6,331 and 5,091 DEL, respectively, were identified from the 1-bp INDELs (**Figure 3**). In the CDS regions of ‘Nagafu No. 2’ and ‘Qinguan,’ 509 and 414 INS, respectively, and 841 and 678 DEL, respectively, were identified from the 1-bp INDELs (**Figure 3**). Detailed distribution information on INDELS of other sizes is shown in **Figure 3**. We identified 171,520 non-synonymous SNPs in 23,111 genes for ‘Nagafu No. 2’ and 147,090 non-synonymous SNPs in 21,400 genes for ‘Qinguan’ apple varieties, but there were relatively small numbers of frameshift INDELs within the genes of both apple varieties (**Supplementary Table S5**). We also analyzed the distribution of the SNPs in Pfam-containing genes, and the range of the ratio of non-synonymous-to-synonymous SNPs (ns/s SNPs) was 0.48 to 1.4 in these genes, indicating that the Pfam domains may have more amino acid substitutions in the genome (**Figure 4**).

The numbers of SNPs, non-synonymous SNPs, SVs, and frameshifts associated with the genes in both ‘Nagafu No. 2’ and ‘Qinguan’ apple varieties can be found in Additional file 2. Additionally, detailed information on the functional and annotation-related information from databases such as Swissprot, Nr, COG, GO, and KEGG for the SNPs, non-synonymous SNPs, SVs, and frameshifts in ‘Nagafu No. 2’ and ‘Qinguan’ apple varieties can be found in Additional file 3. The details of the GO function analysis for genes with SNPs, non-synonymous SNPs, SVs, and INDELs in the two varieties can be seen in Additional files 4, 5, 6, and 7. For example, the GO terms significantly enriched for genes with SNPs and non-synonymous SNPs in both ‘Nagafu No. 2’ and ‘Qinguan’ apple varieties were mainly involved in the defense response (GO:0006952), signal transduction (GO:0007165), arginine catabolic process (GO:0006527), protein targeting to membrane (GO:0006612), and ethylene biosynthetic process (GO:0009693) categories (*P*-value <0.01; Additional files 4 and 5). However, the genes with INDELs were mainly involved in sex determination (GO:0007530), succinate metabolic process (GO:0006105), regulation of ion transport (GO:0043269), response to herbivore (GO:0080027), positive regulation of flavonoid biosynthetic process (GO:0009963), and mitochondrial transport (GO:0006839; *P*-value <0.01; Additional file 6), suggesting that the biological functions of genes with different genome variations had significantly different effects on the phenotypes of these apple varieties.

Meanwhile, the KEGG analysis of the genes with DNA polymorphisms also identified particularly enriched pathways (Additional files 8, 9, 10 and 11). Among them, the different genes with SNPs were enriched in 104 KEGG pathways, and 11 pathways were significantly enriched, including plant hormone signal transduction (ko04075), circadian rhythm (ko04710), photosynthesis (ko00195), and glycerophospholipid metabolism (ko00564; *P*-value <0.1; Additional file 8). Genes with non-synonymous SNPs were significantly enriched in 12 pathways, including vitamin B6 metabolism (ko00750), plant hormone signal transduction (ko04075), and oxidative phosphorylation (ko00190; *P*-value <0.1; Additional file 9). The genes with INDELs were enriched in 72 KEGG pathways, but only four pathways were significantly enriched: ribosome (ko03010), vitamin B6 metabolism (ko00750), sulfur metabolism (ko00920), and mRNA surveillance pathway (ko03015; *P*-value <0.1; Additional file 10).

### Genetic Variation between Easy-Flowering and Difficult-Flowering Apple Varieties

**FIGURE 5:**
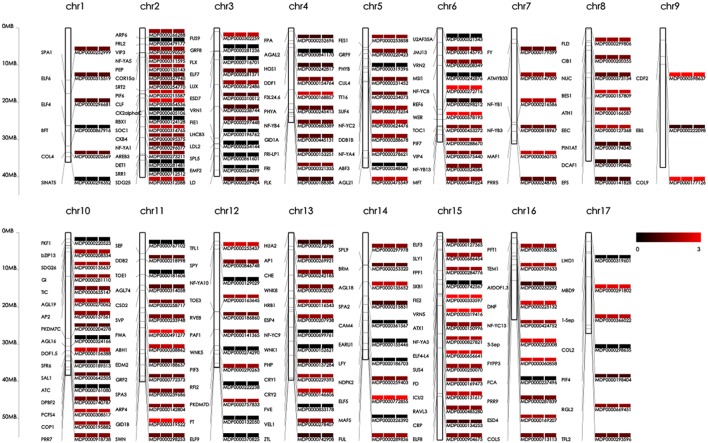
**Genetic linkage maps of ‘Nagafu No. 2,’ ‘Qinguan,’ and ‘Golden Delicious’ constructed using 190 flowering genes.** The abbreviations for the flowering genes are shown on the left and the mutation indices of ‘Nagafu No. 2,’ ‘Qinguan,’ and ‘Golden Delicious’ are shown on the right. The mutation indices were obtained by SNP numbers in flowering genes at log10 among the three apple varieties. The corresponding values are shown as colors from black (0) to red (3).

We speculated that some of the identified genetic variations might contribute to the phenotypic differences in floral-associated traits; therefore, we focused our analysis on SNPs, SVs, and INDELs associated with flowering genes in genic regions. We used the gene set from the reference ‘Golden delicious’ genome as the control and identified all of the shared variations involved in flowering genes between the difficult-flowering ‘Nagafu No. 2’ and easy-flowering ‘Qinguan’ apple varieties. Genetic linkage maps of ‘Nagafu No. 2,’ ‘Qinguan,’ and ‘Golden Delicious’ were also constructed in association with the 190 flowering genes (**Figure 5**). The distribution of flowering genes was significantly different among the 17 chromosomes within each variety (**Figure 5**). For example, 18 flowering genes were found on chromosome 10, but there were just three on chromosome 9 (**Figure 5**). In addition, the locations and distributions of these flowering genes on each chromosome showed large differences among the varieties (for the details, see **Figure 5**).

### Identification by qRT-PCR of Flowering Genes in Leaves and Buds

**FIGURE 6:**
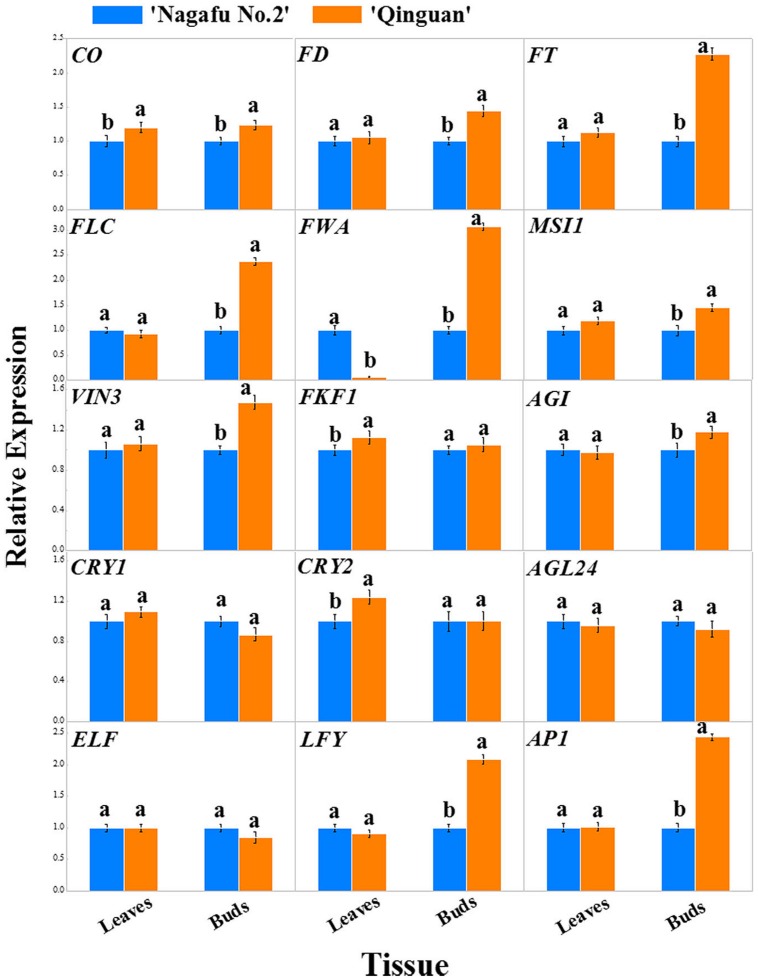
**Expression profiles of flowering genes associated with the multiple pathways in leaves and buds of ‘Nagafu No. 2’ and ‘Qinguan’ apples.** Note: *CO, CONSTANS; FT, FLOWERING LOCUS T; FKF1, FLAVIN-BINDING KELCH REPEAT F BOX 1; ELF3, EARLY FLOWERING 3; CRY1, CRYPTOCHROME 1; CRY2, CRYPTOCHROME 2; AP1, APETALA1; VIN3, VERNALIZATION INSENSITIVE 3; FLC, FLOWERING LOCUS C; MSI1, MULTICOPY SUPRESSOR OF IRA1; FD; LFY; AGL24, AGAMOUS-LIKE 24; GAI, GIBBERELLIC ACID INSENSITIVE; FWA, FLOWERING WAGENINGEN.*

**FIGURE 7:**
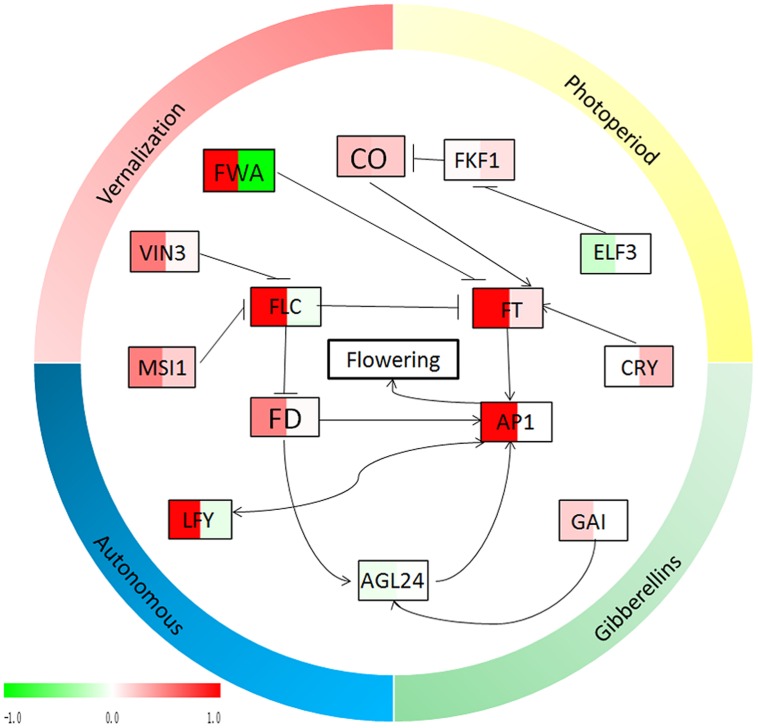
**Relationship diagram of the flowering genes from **Figure 5**.** The expression profiles of the flowering genes, which were calculated by the relative expressions transformed by log 2 (‘Qinguan’/‘Nagafu No. 2’), are indicated by color, from green (-1) to red (1). In each frame, the left side indicates the relative expression levels in buds and the right side indicates the relative expression levels in leaves. Green indicates that the relative expression in ‘Qinguan’ is lower than in ‘Nagafu No. 2,’ and red indicates that the relative expression in ‘Qinguan’ is higher than in ‘Nagafu No. 2.’

The expression profiles of 15 flowering genes, which were mainly involved in four major flowering promotion pathways, photoperiod, autonomous, vernalization, and gibberellin, were identified in leaves and buds of two apple varieties using RT-PCR (**Figures 6** and **7**). In this study, the *CO*, *FT*, *CRY*, and *FKF1* genes, which are related to photoperiod, showed higher expression levels in the leaves and buds of ‘Qinguan’ than of ‘Nagafu No. 2’ (**Figure 6**). The *FWA* expression level in the buds of ‘Qinguan’ was significantly higher than in ‘Nagafu No. 2’; however, its expression level in the leaves of ‘Qinguan’ was significantly lower than in the leaves of ‘Nagafu No. 2’ (**Figure 6**). The vernalization pathway-related genes *VIN3* and *MSI1* were expressed significantly higher in the leaves and buds of ‘Qinguan’ than in those of ‘Nagafu No. 2’ (**Figure 6**). *FLC* plays a key role in the autonomous pathway, and its expression in the buds of ‘Qinguan’ was significantly higher than in ‘Nagafu No. 2’; however, in leaves, its expression was significantly higher in ‘Nagafu No. 2’ (**Figure 6**). In the gibberellin pathway, the expression of *GAI* in ‘Qinguan’ was significantly higher than in ‘Nagafu No. 2,’ but the downstream gene, *AGL24*, showed the reverse expression pattern (**Figure 6**). In addition, a regulatory relationship diagram of these flowering genes in different pathways, as well as models of their expression profiles in leaves and buds between difficult-flowering apple variety ‘Nagafu No. 2’ and easy-flowering apple variety ‘Qinguan,’ are shown in **Figure 7**.

### Isolation of the Upstream Regulatory Region of the Md*FT* Gene from ‘Nagafu No. 2’ and ‘Qinguan’

We cloned and amplified the *FT* promoter region and obtained 1,622-bp and 1,624-bp sequences from ‘Nagafu No. 2’ and ‘Qinguan’ apple leaves, respectively. The two sequences of the *FT* promoter were very similar: 93.1% of the promoter sequence was identical; however, the remaining 6.9% of the sequence was significantly different and included deletions, conversions, and inversions (**Supplementary Figure S6**).

## Discussion

Bioinformatics tools and the rapid development of sequencing technologies have made the complete genome sequencing of many perennial fruit crops possible, providing a starting point to unravel the genetic variation and diversity existing on the genome scale (Jaillon et al., 2007; Velasco et al., 2010; Verde et al., 2013; Wu et al., 2013; Xu et al., 2013). Genome-wide patterns of genetic variation were then captured by sampling a relatively small number of genomes. The whole-genome re-sequencing of two elite apple varieties ‘Nagafu No. 2’ and ‘Qinguan,’ and their comparisons with the reference ‘Golden delicious’ genome sequence (Velasco et al., 2010), allowed us to comprehensively survey SNPs and SVs. DNA polymorphisms on a genome-wide scale were identified, revealing a high level of genetic diversity between ‘Nagafu No. 2’ and ‘Qinguan’ Our results also showed how NGS technologies can be powerful tools for studying genome-wide DNA polymorphisms, querying genetic diversity, and enabling molecular-based improvements of apple breeding.

SNP markers have been employed widely to study evolutionary relationships, population structures, and association analyses in rice, peach, and pear (McNally et al., 2009; Parida et al., 2012; Bai H. et al., 2013; Lyu et al., 2013; Montanari et al., 2013; Cao et al., 2014). However, only a few studies have used genome-wide SNP markers to understand the structure and diversity in different genomes of Malus species (Chagné et al., 2008, 2012). In this study, ‘Nagafu No. 2’ had more SNPs than ‘Qinguan,’ which indicated that ‘Qinguan’ is more closely related to ‘Golden Delicious’ than ‘Nagafu No. 2’ (**Supplementary Table S3**). This is unsurprising because ‘Qinguan’ is a hybrid progeny of ‘Golden Delicious’ and ‘Cockscomb.’ A similar phenomenon was reported in rice (Bai S.L. et al., 2013). In addition, our results indicated that re-sequencing could be a good experimental tool to better understand evolutionary relationships in plant species. More genotypes are needed to produce a comprehensive understanding of the complex relationships and evolution in the Malus species.

The average proportions of genic SNPs, exons, introns, or intergenic, were similar in the ‘Nagafu No. 2’ and ‘Qinguan’ varieties (**Table 3**), and were similar to those in peach (Cao et al., 2014). Compared with *Arabidopsis* (Clark et al., 2007), rice (Feltus et al., 2004), and *Miscanthus sinensis* (Clark et al., 2014), the intergenic regions of apple genes harbor more SNPs, which might be related to the increased size of the intergenic regions in the apple genome (**Table 3**). The proportions of genic SVs were also similar between the two varieties (**Table 3**). The difference in genetic diversity between ‘Nagafu No. 2’ and ‘Qinguan,’ as well as the difference in the distribution of this diversity in different genetic regions, may involve changes in agronomic and floral-associated traits between the two apple varieties.

**Table 3:** Annotation of SNPs identified between samples and the reference genome.

		Exon		Intron		Intergenic	
				
Variety	Type	Number	percentage	Number	percentage	Number	percentage
‘Nagafu No.2’	SNP	280,835	10.13%	315,948	11.4%	2,174,946	78.47%
	SV	3,963	5.0%	19,659	24.75%	55,798	70.26%
‘Qinguan’	SNP	242,328	10.71%	247,806	10.94%	1,773,297	78.35%
	SV	3,196	5.23%	15,072	24.64%	42,895	70.13%


*SNPs and SVs were classified as genetic and intergenic, and locations within the gene models were annotated based on the annotations of the reference genome.*

In this study, we identified genetic variation, including large numbers of SNPs, SVs, and INDELs, in the two samples when compared with the ‘Golden Delicious’ reference genome. The distributions of these polymorphisms were uneven within the chromosomes. A similar study on peach (*Prunus persica*) showed multiple variation patterns and an uneven distribution of the variation across the genome (Cao et al., 2014), as did a study on *Sorghum bicolor* (Zheng et al., 2011). Indeed, many studies, for example, in rice (Caicedo et al., 2007; McNally et al., 2009; Subbaiyan et al., 2012), soybean (Kim et al., 2010), and peach (Cao et al., 2014), have shown that SNPs, SVs, and INDELs on each chromosome are unevenly distributed and are often concentrated in certain chromosomal regions. Meanwhile, in our results, chromosome 15 had the highest number of SNPs and chromosome 16 had the lowest number of SNPs in both cultivars. Moreover, for chromosome 1, ‘Nagafu No. 2’ had more SNPs and SVs than ‘Qinguan,’ which suggested that these different distribution types and features of variation may contribute to the differences in certain characteristics, such as flowering and resistance, between the two apple varieties. In addition, the distribution of SNPs within chromosomes is non-random; for example, a high SNP density was observed between 8.7 and 9.2 Mb on chromosome 8, but no SNPs were found in a longer interval of 200 kb, from 27.2 to 27.4 Mb, on chromosome 11 (Subbaiyan et al., 2012). A similar result was observed in our study (**Supplementary Figure S5**).

Our results showed that the number of INDELs of different lengths (1–3 bp) in the CDS regions and the whole genome were significantly different between ‘Nagafu No. 2’ and ‘Qinguan,’ and similar results have been seen in other plants (McNally et al., 2009; Subbaiyan et al., 2012). Compared with the high numbers of non-synonymous SNPs in ‘Nagafu No. 2’ and ‘Qinguan,’ relatively few frameshift INDELs within genes were observed, which was similar to studies in tomato (Hirakawa et al., 2013), rice (Parida et al., 2012), and apple (Chagné et al., 2012). Genome variations, such as SNPs (non-synonymous or synonymous), INDELs, and SVs, at the whole-genome level in the two apple varieties, which result in amino acid changes, were mainly caused by positive selection during adaptation to environmental changes during the evolutionary process.

The two varieties and ‘Golden Delicious,’ which is also easy to flower and the flowering rate in 6-year-old trees is ∼65%, have significant differences in floral characteristics; therefore, we constructed genetic linkage maps of flowering genes in the three varieties and compared the genetic variation of these genes across the whole genome. The genome variation of these flowering genes showed significant differences among the three apple varieties (**Figure 5**), which may explain the differences in flowering-related traits among them. Additionally, genomic variation, such as large-effect SNPs, result in changes to biological characteristics, including growth characteristics, plant phenotypes, and the resistance responses (Zheng et al., 2011). Similar results have been reported in other plants (Parida et al., 2012; Clark et al., 2014). In addition, to determine the functions of genes with DNA polymorphisms between ‘Nagafu No. 2’ and ‘Qinguan,’ we performed GO and KEGG analyses of genes with SNPs, non-synonymous SNPs, SVs, and INDELs, in the two varieties (Additional files 4, 5, 6, 7, 8, 9, 10, and 11). This analysis focused on key genes with DNA polymorphisms in the two apple varieties, so that we can more accurately understand the relationships between these genes with DNA polymorphisms and the phenotypic traits.

Previous studies have shown that there are four major flowering pathways photoperiod, autonomous, vernalization, and gibberellin, which are associated with complex gene regulation process (Bernier and Périlleux, 2005; Amasino, 2010; Koutinas and Pepelyankov, 2010; Turnbull, 2011; Bernier, 2013). The photoperiod and vernalization pathways process environmental signals to the floral transition, whereas the autonomous and gibberellin pathways act independently of external signals (Mutasa-Gottgens and Hedden, 2009). The photoreceptor, circadian clock, and circadian clock-regulated genes constitute the photoperiod pathway and act in response to a long photoperiod (Searle and Coupland, 2004; Jung et al., 2007; Fornara and Coupland, 2009). In our study, the photoperiod genes *CO* and *FT* were significantly more highly expressed in the leaves and buds of ‘Qinguan’ compared with ‘Nagafu No. 2.’ The relatively higher expression levels of these flowering control genes associated with the photoperiod in plants can significantly promote flower bud formation (Gould et al., 2013; Fan et al., 2014). In addition, in the vernalization pathway, the *VIN3* and *MSI1* genes showed higher expression levels in ‘Qinguan’ and in buds. In addition, *FLC* plays a key role in the autonomous pathway (Liu et al., 2007; Porto et al., 2015). In the present study, *FLC* expression in the buds of ‘Qinguan’ was much higher than in those of ‘Nagafu No. 2’; however, in leaves, its expression was lower than in ‘Nagafu No. 2.’ The expression profiles of these key flowering genes were significantly associated with bud growth, floral induction, and physiological phenotypic characteristics involved in flowering in plants (Jean Finnegan et al., 2011; Ream et al., 2014). Gibberellins play key roles in the regulation of flower induction as part of the complex floral regulatory networks (Mutasa-Gottgens and Hedden, 2009) and in multiple flowering pathways (Liu et al., 2008; Wilkie et al., 2008; Lee and Lee, 2010). Indeed, of the gibberellin pathway genes, the expression of *GAI* in ‘Qinguan’ buds was significantly higher than in ‘Nagafu No. 2’ buds; however, the downstream gene *AGL24* showed the reverse pattern, suggesting that differences in the expression levels of genes associated with multiple flowering pathways may contribute to differences in floral-associated traits between ‘Nagafu No. 2’ and ‘Qinguan.’ Meanwhile, the expression profiles of key flowering genes in apple buds and leaves in the four major flowering pathways suggested that the photoperiod and autonomous flowering pathways make a major contribution to the differences in the floral-associated traits of two apple varieties (**Figure 7**).

Sequence variation in promoter regions mediates a transcriptional regulation mechanism associated with anthocyanin biosynthesis genes in grape (Kobayashi et al., 2004) and apple (Espley et al., 2009). In our study, we speculated that some of the identified genetic variation might contribute to the phenotypic differentiation of floral-associated traits. We focused our analysis on SNPs, INDELs, and SVs in promoter regions between ‘Nagafu No. 2’ and ‘Qinguan.’ As a result, we amplified the *FT* promoter region from ‘Nagafu No. 2’ and ‘Qinguan’ to identify and compare the sequence variations between the two varieties. The sequences of the *FT* promoter regions were 93.1% identical, but 6.9%, including deletions, conversions, and inversions, was completely different (**Supplementary Figure S6**), and the expression of the *FT* gene in buds was significantly higher in ‘Qinguan’ than in ‘Nagafu No. 2’ (**Figure 6**). In addition, we predicted transcription factor-binding sites, including ARE, G-Box, GATA-motif, and Spl, in *FT* promoters between the two apple varieties (**Supplementary Table S6**). This suggested that the observed variation may result in different *FT* expression patterns between ‘Nagafu No. 2’ and ‘Qinguan’ apple varieties.

## Conclusion

In the present study, we identified large-scale genomic variation, including SNPs, INDELS, and SVs, among two elite apple varieties ‘Nagafu No 2,’ ‘Qinguan’ and the reference variety ‘Golden Delicious.’ This can be used as a framework for future comparative and functional genomic analyses in fruit trees, as well as providing information for molecular breeding, allele discovery, and agronomic trait screening in apple. ‘Qinguan’ is an elite variety with strong disease resistance and easy flowering, whereas the fruit quality of ‘Nagafu No. 2’ is better than that of other cultivars. Thus, genome-wide comparison studies of variations involved in floral-related traits and resistance responses will also provide a powerful resource to identify key genes and for function-related research. We also established genetic linkage maps of ‘Nagafu No. 2,’ ‘Qinguan,’ and ‘Golden Delicious’ using 190 flowering genes in multiple flowering pathways and compared the distribution characteristics of the genome variations in these genes, which may contribute to a deeper understanding of the differences among a variety of traits across the genome.

## Author Contributions

Conceived and designed the experiment: HM, XL, ZD, and ZC. Performed the experiments: XL, WK, and LY. Analyzed the data: XL, WK, JM, and NA. Developed the model: LX and KW. Wrote the paper: XL, ZD, and SX. Edited the manuscript: AN.

## Conflict of Interest Statement

The authors declare that the research was conducted in the absence of any commercial or financial relationships that could be construed as a potential conflict of interest.

## Footnotes

1 http://faostat.fao.org/

2 http://www.rosaceae.org/

3 http://bio-bwa.sourceforge.net/bwa.shtml

4 http://samtools.sourceforge.net/samtools.shtml

5 https://trac.nbic.nl/Pindel/

6 http://BreakDancer.sourceforge.net/BreakDancermax.html

7 http://www.rosaceae.org/species/malus/malus_x_domestica

8 http://www.ebi.ac.uk/Tools/psa/genewise/

9 http://www.genome.jp/kegg/

10 http://bioinformatics.psb.ugent.be/webtools/plantcare/html/

11 http://simgene.com/Primer3

12 http://www.ncbi.nlm.nih.gov/Traces/sra\_sub/sub.cgi
