# Supplementary material for: Genome-Wide Sequence Variation Identification and Floral-Associated Trait Comparisons Based on the Re-sequencing of the ‘Nagafu No. 2’ and ‘Qinguan’ Varieties of Apple (Malus domestica Borkh.)
Source: Front Plant Sci. 2016 Jun 27;7:908. doi: 10.3389/fpls.2016.00908 (PMC4921462; doi:10.3389/fpls.2016.00908)
Supplement: Supplementary file 2 [file Data_Sheet_2.DOC]

**Identifying** **genome-wide sequence variations and comparing floral-associated traits based on re-sequencing of two varieties of apple *(******Malus domestica Borkh.)* ‘Nagafu No. 2’ and ‘Qinguan’**

**Short title: Sequence variations and floral traits in apple**

**Libo Xing†, Dong Zhang†, Xiaomin Song†, Kai Weng, Yawen Shen, Youmei Li, Caiping Zhao, Juanjuan Ma, Na An, Mingyu Han***

**Supplementary data**

**Supplementary File 1**

**Fig. S1.** Detailed information of data quality, including base distribution, cycle average phred score, and quality distribution in ‘Nagafu No. 2’ (a, b, and c) and ‘Qinguan’ (d, e, and f) apple varieties.

**Fig. S2.** The depth distribution of ‘Nagafu No. 2’ and ‘Qinguan’ apple varieties.

**Fig. S3.** The insert size distribution of ‘Nagafu No. 2’ and ‘Qinguan’ apple varieties.

**Fig. S4.** The detail information of the depth distribution on 17 chromosomes in both of ‘Nagafu No. 2’ (a) and ‘Qinguan’ (b) apple varieties.

**Fig. S5.** Distribution of single nucleotide polymorphisms (SNPs) and SVs between ‘Nagafu No. 2’ (a) and ‘Qinguan’ (b) apple varieties in the 17 chromosomes. The x-axis represents the physical distance along each chromosome, split into 100 kb windows. The total size of each chromosome is shown in brackets. The y-axis indicates the number of SNPs (left, red lines) and SVs (right, blue lines). The total SNP and SV numbers in each chromosome are shown in parentheses.

**Fig. S6.** The promoter sequence of the *FT* gene and some DNA polymorphism between ‘Nagafu No. 2’ and ‘Qinguan’ apple varieties.

**Table S1.** List of primers used in this study.

**Table S2.** Number and densities of SNPs, SVs, and INDELs detected between samples and the reference genome.

**Table S3.** Classification of nucleotide substitutions in the SNPs detected in ‘Nagafu No. 2’ and ‘Qinguan’.

**Table S4.** Number and densities of SVs detected between samples and the reference genome.

**Table S5.** The number of SNPs, non-synonymous SNPs, SVs, and INDELs with corresponding genes.

**Table S6.** The *cis*-elements in *FT* gene promoter sequences between ‘Nagafu No. 2’ and ‘Qinguan’ apple varieties.

**Supplementary File 2**

**Additional file 1.** The detected SVs in 17 chromosomes and their detail information in both ‘Nagafu No. 2’ and ‘Qinguan’ apple varieties.

**Additional file 2.** The number of DNA Polymorphism including SNPs, non-SNPs, SVs, and frameshifts associated with genes in both ‘Nagafu No. 2’ and ‘Qinguan’ apple varieties.

**Additional file 3.** The detailed annotation information of genes with the DNA polymorphism including SNPs, non-SNPs, SVs, and frameshifts, and their functional analysis in database such as Swissprot, COG, GO, Nr, and KEGG in both ‘Nagafu No. 2’ and ‘Qinguan’ apple varieties.

**Additional file 4.** GO analysis of differently genes with SNPs variation in both ‘Nagafu No. 2’ and ‘Qinguan’ apple varieties.

**Additional file 5.** GO analysis of differently genes with non-synonymous SNPs variation in both ‘Nagafu No. 2’ and ‘Qinguan’ apple varieties.

**Additional file 6.** GO analysis of differently genes with INDEL variation in both ‘Nagafu No. 2’ and ‘Qinguan’ apple varieties.

**Additional file 7.** GO analysis of genes with SVs variation in both ‘Nagafu No. 2’ and ‘Qinguan’ apple varieties.

**Additional file 8.** KEGG pathway analysis of differently genes with SNPs variation in both ‘Nagafu No. 2’ and ‘Qinguan’ apple varieties.

**Additional file 9.** KEGG pathway analysis of differently genes with non-synonymous SNPs variation in both ‘Nagafu No. 2’ and ‘Qinguan’ apple varieties.

**Additional file 10.** KEGG pathway analysis of differently genes with INDEL variation in both ‘Nagafu No. 2’ and ‘Qinguan’ apple varieties.

**Additional file 11.** KEGG pathway analysis of genes with SVs variation in both ‘Nagafu No. 2’ and ‘Qinguan’ apple varieties.

**Supplementary data files**

**Table S1.** List of primers used in this study.

| **Gene** | **Gene ID** | **Forward primer** | **Reverse primer** |
| --- | --- | --- | --- |
| ***AP1*** | MDP0000269921 | AAAGGAGAAGGAGAAGGCTGCA | TGTGGAAGCAGGTCAAGGTCAT |
| ***CRY2*** | MDP0000146606 | TTGGCTAGAGAACGCTTGACGA | ACTTCATCTGTGCCGTTTGAGC |
| ***FT*** | MDP0000132050 | GCCAGCGAGGTTTCAACTTCTT | TGCCGCAGTAGTTGCTGGAATA |
| ***FLC*** | MDP0000326390 | ACAAGTGTGCAAGCTCCAGAAG | GGAAGCCGAGTTTGCTGAATGT |
| ***LFY*** | MDP0000186703 | AGAAGCAGCGGAGGATGAAGAA | GTCATGTCGTCCATGTCGTTGT |
| ***CRY1*** | MDP0000229393 | TGGCTCATCTTGATTGCTCCCT | TGGCACCAGTGGAAACAACAAC |
| ***FD*** | MDP0000169473 | AGTGACCAGACCAACCACAACA | ATTTGGGTGGTGGGATCAGTGA |
| ***VIN3*** | MDP0000319416 | ACGGACATTGTCTCGTGTGGAT | ATTGGACATGCCGGTGTAGTGA |
| ***CO*** | MDP0000298635 | GTTGTTGCTCAACCCGGTGAAA | ATTGTTGTTGCTGCTGCTGGT |
| ***GAI*** | MDP0000669451 | GGTTCACGGAGGCATTGCATTA | TGCCCAAATACACCTCGGACAT |
| ***AGL24*** | MDP0000233948 | TGAAGGTGGCGGAGAAATCTGA | TCGTAGTCTTGAGGACCGTTGT |
| ***MSI1*** | MDP0000242876 | CATGGGCAAAGACGACGAAGAA | AAGCCACTCCACAGTAAGCGAT |
| ***FWA*** | MDP0000491277 | GATGTGACGAGAGTGGCTGAGA | TGTCGGTGGTAACGCTTCTTCT |
| ***FKF1*** | MDP0000220523 | AAGGCGGCATCCTCTTGTAGAT | TGCCTATGATGTGAGTGACGGT |
| ***ELF3*** | MDP0000127365 | TCAACTCCGATGACAGGCAACT | AAACGGGTCCAGCAAACCAATG |

**Table S2.** Number and densities of SNPs, SVs, and INDELs detected between samples and the reference genome

| **Chromosome** | | | **Category** | **SNPs** | | **SVs** | | **INDELs** | |
| --- | --- | --- | --- | --- | --- | --- | --- | --- | --- |
|  |  |  |  | **‘Nagafu No.2’** | **‘Qinguan’** | **‘Nagafu No.2’** | **‘Qinguan’** | **‘Nagafu No.2’** | **‘Qinguan’** |
| **chr1** | | Number | | 133130 | 115431 | 4016 | 3245 | 75285 | 63706 |
|  |  | Density | | (3689.4) | (3198.9) | (111.3) | (89.9) | (2086.3) | (1765.5) |
| **chr2** | | Number | | 218178 | 162624 | 6248 | 4567 | 124383 | 94712 |
|  |  | Density | | (5431.0) | (4048.1) | (155.5) | (113.7) | (3096.2) | (2357.6) |
| **chr3** | | Number | | 185031 | 144395 | 5335 | 4054 | 108039 | 84514 |
|  |  | Density | | (4636.5) | (3618.2) | (133.7) | (101.6) | (2707.2) | (2117.7) |
| **chr4** | | Number | | 118720 | 110726 | 3495 | 2933 | 70139 | 65616 |
|  |  | Density | | (4671.8) | (4357.2) | (137.5) | (115.4) | (2760.1) | (2582.1) |
| **chr5** | | Number | | 159418 | 137138 | 4710 | 3822 | 90438 | 80623 |
|  |  | Density | | (4239.4) | (3646.9) | (125.3) | (101.6) | (2405.0) | (2144.0) |
| **chr6** | | Number | | 123261 | 114899 | 3696 | 3091 | 69901 | 66607 |
|  |  | Density | | (4018.9) | (3746.2) | (120.5) | (100.8) | (2279.1) | (2171.7) |
| **chr7** | | Number | | 143666 | 128747 | 3832 | 3148 | 83527 | 75703 |
|  |  | Density | | (4607.5) | (4129.0) | (122.9) | (101.0) | (2678.8) | (2427.9) |
| **chr8** | | Number | | 162893 | 109511 | 4509 | 3300 | 94396 | 63746 |
|  |  | Density | | (4550.0) | (3058.9) | (125.9) | (92.2) | (2636.7) | (1780.6) |
| **chr9** | | Number | | 175621 | 162728 | 4973 | 4224 | 103581 | 97393 |
|  |  | Density | | (4681.4) | (4337.8) | (132.6) | (112.6) | (2761.1) | (2596.2) |
| **chr10** | | Number | | 176550 | 127429 | 5752 | 3816 | 95884 | 68990 |
|  |  | Density | | (4599.0) | (3319.4) | (149.8) | (99.4) | (2497.7) | (1797.1) |
| **chr11** | | Number | | 226778 | 152490 | 6535 | 4442 | 125204 | 85105 |
|  |  | Density | | (5655.7) | (3803.0) | (163.0) | (110.8) | (3122.5) | (2122.5) |
| **chr12** | | Number | | 163207 | 124596 | 5167 | 3921 | 90058 | 70422 |
|  |  | Density | | (4499.0) | (3434.6) | (142.4) | (108.1) | (2482.5) | (1941.3) |
| **chr13** | | Number | | 173473 | 152352 | 5573 | 4371 | 95896 | 82331 |
|  |  | Density | | (4371.1) | (3838.9) | (140.4) | (110.1) | (2416.4) | (2074.6) |
| **chr14** | | Number | | 144945 | 121928 | 4533 | 3466 | 79807 | 67889 |
|  |  | Density | | (4243.6) | (3569.7) | (132.7) | (101.5) | (2336.5) | (1987.6) |
| **chr15** | | Number | | 231577 | 193484 | 6817 | 5475 | 131976 | 109667 |
|  |  | Density | | (4151.9) | (3469.0) | (122.2) | (98.2) | (2366.2) | (1966.2) |
| **chr16** | | Number | | 104010 | 99017 | 3448 | 2786 | 60055 | 56509 |
|  |  | Density | | (4432.9) | (4220.1) | (147.0) | (118.7) | (2559.6) | (2408.4) |
| **chr17** | | Number | | 130671 | 105393 | 4024 | 3103 | 74234 | 60527 |
|  |  | Density | | (4817.8) | (3885.8) | (148.4) | (114.4) | (2737.0) | (2231.6) |
| **Total** | | Number | | 2771129 | 2262888 | 82663 | 63764 | 1572803 | 1294060 |
|  |  | Density | | (4546.9) | (3746.0) | (135.9) | (105.3) | (2584.1) | (2145.4) |

**Note:** Density, the polymorphisms number/physical distance of each chromosomes ratio

**Table S3.** Classification of nucleotide substitutions in the SNPs detected in ‘Nagafu No. 2’ and ‘Qinguan’

| **Chromosome** | **Transition** | | **Transversion** | | | **Heterozygosity** | |
| --- | --- | --- | --- | --- | --- | --- | --- |
|  | **‘Nagafu No.2’** | **‘Qinguan’** | **‘Nagafu No.2’** | **‘Qinguan’** | **‘Nagafu No.2’** | | **‘Qinguan’** |
| **chr1** | 68.13% | 68.28% | 31.87% | 31.72% | 81.45% | | 90.78% |
|  | (2513.6) | (2184.2) | (1175.8) | (1014.7) | (3005.0) | | (2903.9) |
| **chr2** | 68.33% | 68.15% | 31.67% | 31.85% | 74.09% | | 88.07% |
|  | (3711.0) | (2758.8) | (1720.0) | (1289.3) | (4023.8) | | (3565.2) |
| **Chr3** | 68.71% | 68.27% | 31.29% | 31.73% | 78.18% | | 91.38% |
|  | (3185.7) | (2470.2) | (1450.8) | (1148.1) | (3624.8) | | (3306.3) |
| **Chr4** | 68.09% | 68.29% | 31.91% | 31.71% | 85.53% | | 92.70% |
|  | (3181.0) | (2975.6) | (1490.8) | (1381.7) | (3995.8) | | (4039.2) |
| **Chr5** | 67.59% | 67.71% | 32.41% | 32.29% | 83.82% | | 91.67% |
|  | (2865.4) | (2469.3) | (1374.0) | (1177.6) | (3553.5) | | (3343.1) |
| **Chr6** | 68.31% | 68.40% | 31.69% | 31.60% | 81.00% | | 89.30% |
|  | (2745.3) | (2562.4) | (1273.6) | (1183.8) | (3255.3) | | (3345.4) |
| **Chr7** | 68.12% | 68.02% | 31.88% | 31.98% | 89.66% | | 93.67% |
|  | (3138.6) | (2808.5) | (1468.9) | (1320.5) | (4131.1) | | (3867.6) |
| **Chr8** | 68.64% | 68.30% | 31.36% | 31.70% | 82.12% | | 86.08% |
|  | (3123.1) | (2089.2) | (1426.9) | (969.7) | (3736.4) | | (2633.1) |
| **Chr9** | 67.98% | 68.27% | 32.02% | 31.73% | 87.11% | | 90.61% |
|  | (3182.5) | (2961.4) | (1499.0) | (1376.4) | (4078.0) | | (3930.5) |
| **Chr10** | 67.66% | 67.45% | 32.34% | 32.55% | 75.17% | | 90.90% |
|  | (3111.7) | (2239.0) | (1487.3) | (1080.5) | (3457.1) | | (3017.4) |
| **Chr11** | 67.78% | 67.90% | 32.22% | 32.10% | 81.88% | | 91.16% |
|  | (3833.4) | (2582.2) | (1822.3) | (1220.8) | (4630.9) | | (3466.8) |
| **Chr12** | 68.05% | 67.58% | 31.95% | 32.42% | 78.52% | | 86.76% |
|  | (3061.6) | (2321.1) | (1437.4) | (1113.5) | (3532.6) | | (2979.9) |
| **Chr13** | 68.58% | 68.42% | 31.42% | 31.58% | 82.53% | | 94.37% |
|  | (2997.7) | (2626.6) | (1373.4) | (1212.3) | (3607.5) | | (3622.8) |
| **Chr14** | 68.64% | 68.61% | 31.36% | 31.39% | 80.06% | | 91.32% |
|  | (2912.8) | (2449.2) | (1330.8) | (1120.5) | (3397.4) | | (3259.9) |
| **Chr15** | 68.51% | 68.27% | 31.49% | 31.73% | 80.21% | | 88.49% |
|  | (2844.5) | (2368.3) | (1307.4) | (1100.7) | (3330.3) | | (3069.7) |
| **Chr16** | 68.09% | 68.51% | 31.91% | 31.49% | 85.28% | | 93.89% |
|  | (3018.4) | (2891.2) | (1414.6) | (1328.9) | (3780.4) | | (3962.3) |
| **Chr17** | 67.79% | 67.65% | 32.21% | 32.35% | 82.01% | | 91.68% |
|  | (3266.0) | (2628.7) | (1551.8) | (1257.1) | (3951.1) | | (3562.5) |
| **Average** | 68.19% | 68.12% | 31.81% | 31.88% | 81.29% | | 90.69% |
|  | (3101.2) | (2529.9) | (1446.7) | (1184.0) | (3697.0) | | (3368.1) |

**Note:** Chr (chromosome), Transition (C/T and G/A), Transversion (C/G, T/A, A/C, and G/T), Heterozygosity (two or more different alleles of the same gene). The number in brackets means the densities of transition, transversion, and heterozygosity on each chromosome per Mb.

**Table S4**. Number and densities of SVs detected between samples and reference genome.

| **Chromosome** | **Category** | **SVs** |  | **INS** |  | **DEL** |  | **IDE** |  | **INV** |  | **ITX** |  | **CTX** |  |
| --- | --- | --- | --- | --- | --- | --- | --- | --- | --- | --- | --- | --- | --- | --- | --- |
|  |  | **Nagafu No.2** | **Qinguan** | **Nagafu No.2** | **Qinguan** | **Nagafu No.2** | **Qinguan** | **Nagafu No.2** | **Qinguan** | **Nagafu No.2** | **Qinguan** | **Nagafu No.2** | **Qinguan** | **Nagafu No.2** | **Qinguan** |
| **Chr1** | Percetage | 4.86% | 5.09% | 38.20% | 39.32% | 56.55% | 55.19% | 0.60% | 0.49% | 0.10% | 0.06% | 0.40% | 0.37% | 4.16% | 4.56% |
|  | Density | (111.31) | (89.94) | (42.51) | (34.35) | (62.94) | (49.63) | (0.67) | (0.44) | (0.11) | (0.05) | (0.45) | (0.33) | (4.63) | (4.10) |
| **Chr2** | Percetage | 7.56% | 7.16% | 38.97% | 37.07% | 57.01% | 58.24% | 0.66% | 0.50% | 0.05% | 0.02% | 0.32% | 0.35% | 2.99% | 3.81% |
|  | Density | (155.54) | (113.69) | (60.61) | (44.30) | (88.67) | (66.21) | (1.03) | (0.57) | (0.08) | (0.02) | (0.50) | (0.40) | (4.65) | (4.33) |
| **Chr3** | Percetage | 6.45% | 6.36% | 38.59% | 37.62% | 58.39% | 58.53% | 0.52% | 0.59% | 0.07% | 0.10% | 0.34% | 0.37% | 2.08% | 2.79% |
|  | Density | (133.68) | (101.58) | (51.59) | (39.20) | (78.06) | (59.46) | (0.70) | (0.60) | (0.09) | (0.10) | (0.45) | (0.38) | (2.78) | (2.83) |
| **Chr4** | Percetage | 4.23% | 4.60% | 37.57% | 37.13% | 59.08% | 59.26% | 0.72% | 0.75% | 0.09% | 0.17% | 0.46% | 0.41% | 2.09% | 2.28% |
|  | Density | (137.54) | (115.43) | (51.67) | (43.36) | (81.25) | (68.40) | (0.99) | (0.87) | (0.12) | (0.20) | (0.63) | (0.47) | (2.87) | (2.63) |
| **Chr5** | Percetage | 5.70% | 5.99% | 40.70% | 38.10% | 56.73% | 58.79% | 0.62% | 0.65% | 0.04% | 0.13% | 0.30% | 0.47% | 1.61% | 1.86% |
|  | Density | (125.27) | (101.65) | (50.98) | (41.37) | (71.06) | (59.75) | (0.78) | (0.66) | (0.05) | (0.13) | (0.38) | (0.48) | (2.02) | (1.89) |
| **Chr6** | Percetage | 4.47% | 4.85% | 40.61% | 39.60% | 56.71% | 57.97% | 0.84% | 0.61% | 0.00% | 0.00% | 0.27% | 0.36% | 1.57% | 1.46% |
|  | Density | (120.51) | (100.78) | (48.94) | (40.93) | (68.34) | (58.42) | (1.01) | (0.61) | (0.00) | (0.00) | (0.33) | (0.36) | (1.89) | (1.47) |
| **Chr7** | Percetage | 4.64% | 4.94% | 39.38% | 38.95% | 58.14% | 58.80% | 1.04% | 0.73% | 0.05% | 0.13% | 0.23% | 0.51% | 1.15% | 0.89% |
|  | Density | (122.90) | (100.96) | (48.40) | (39.76) | (71.45) | (59.36) | (1.28) | (0.74) | (0.06) | (0.13) | (0.28) | (0.51) | (1.41) | (0.90) |
| **Chr8** | Percetage | 5.45% | 5.18% | 40.16% | 40.06% | 57.95% | 58.21% | 0.82% | 0.61% | 0.13% | 0.09% | 0.51% | 0.33% | 0.42% | 0.70% |
|  | Density | (125.95) | (92.18) | (50.58) | (37.02) | (72.99) | (53.66) | (1.03) | (0.56) | (0.16) | (0.08) | (0.64) | (0.30) | (0.53) | (0.65) |
| **Chr9** | Percetage | 6.02% | 6.62% | 39.65% | 38.80% | 58.98% | 59.85% | 0.92% | 0.95% | 0.16% | 0.14% | 0.28% | 0.26% | 0.00% | 0.00% |
|  | Density | (132.58) | (112.61) | (52.56) | (44.64) | (78.19) | (67.39) | (1.22) | (1.07) | (0.21) | (0.16) | (0.37) | (0.29) | (0.00) | (0.00) |
| **Chr10** | Percetage | 6.96% | 5.98% | 38.04% | 36.43% | 54.31% | 54.14% | 0.66% | 0.52% | 0.10% | 0.13% | 0.50% | 0.63% | 6.38% | 8.15% |
|  | Density | (149.83) | (99.40) | (57.00) | (37.81) | (81.38) | (53.82) | (0.99) | (0.52) | (0.15) | (0.13) | (0.75) | (0.63) | (9.56) | (8.10) |
| **Chr11** | Percetage | 7.91% | 6.97% | 38.03% | 36.22% | 55.21% | 55.81% | 0.67% | 0.50% | 0.02% | 0.00% | 0.35% | 0.50% | 5.72% | 6.98% |
|  | Density | (162.97) | (110.78) | (61.98) | (42.13) | (89.98) | (61.83) | (1.09) | (0.55) | (0.03) | (0.00) | (0.57) | (0.55) | (9.32) | (7.73) |
| **Chr12** | Percetage | 6.25% | 6.15% | 37.41% | 35.53% | 55.18% | 56.52% | 0.66% | 0.56% | 0.08% | 0.10% | 0.62% | 0.61% | 6.06% | 6.68% |
|  | Density | (142.42) | (108.08) | (53.28) | (40.44) | (78.60) | (61.09) | (0.94) | (0.61) | (0.11) | (0.11) | (0.88) | (0.66) | (8.63) | (7.22) |
| **Chr13** | Percetage | 6.74% | 6.85% | 38.17% | 38.30% | 55.28% | 54.04% | 0.70% | 0.82% | 0.04% | 0.02% | 0.34% | 0.43% | 5.47% | 6.38% |
|  | Density | (140.41) | (110.13) | (53.60) | (42.04) | (77.63) | (59.52) | (0.98) | (0.90) | (0.06) | (0.02) | (0.48) | (0.47) | (7.68) | (7.03) |
| **Chr14** | Percetage | 5.48% | 5.44% | 38.19% | 37.48% | 55.06% | 55.68% | 0.68% | 0.61% | 0.09% | 0.00% | 0.31% | 0.35% | 5.67% | 5.89% |
|  | Density | (132.70) | (101.47) | (50.68) | (38.75) | (73.07) | (56.50) | (0.90) | (0.62) | (0.12) | (0.00) | (0.41) | (0.36) | (7.52) | (5.98) |
| **Chr15** | Percetage | 8.25% | 8.59% | 36.39% | 36.31% | 56.99% | 56.68% | 0.84% | 0.68% | 0.06% | 0.07% | 0.37% | 0.46% | 5.35% | 5.81% |
|  | Density | (122.21) | (98.15) | (44.48) | (35.72) | (69.65) | (55.64) | (1.03) | (0.67) | (0.07) | (0.07) | (0.45) | (0.45) | (6.54) | (5.70) |
| **Chr16** | Percetage | 4.17% | 4.37% | 37.70% | 38.16% | 57.74% | 57.07% | 0.64% | 0.54% | 0.03% | 0.00% | 0.26% | 0.43% | 3.63% | 3.80% |
|  | Density | (146.97) | (118.76) | (55.40) | (44.77) | (84.85) | (67.77) | (0.94) | (0.64) | (0.04) | (0.00) | (0.38) | (0.51) | (5.33) | (4.51) |
| **Chr17** | Percetage | 4.87% | 4.87% | 37.40% | 36.58% | 56.81% | 57.43% | 0.82% | 0.61% | 0.12% | 0.00% | 0.47% | 0.39% | 4.37% | 5.00% |
|  | Density | (148.38) | (114.42) | (55.49) | (42.79) | (84.29) | (65.70) | (1.22) | (0.70) | (0.18) | (0.00) | (0.70) | (0.45) | (6.48) | (5.72) |
| **Total** | Percetage | 100% | 100% | 38.47% | 37.64% | 56.71% | 57.13% | 0.72% | 0.63% | 0.07% | 0.07% | 0.38% | 0.43% | 3.65% | 4.10% |
|  | Density | (135.7) | (104.6) | (52.19) | (40.26) | (76.94) | (59.79) | (0.98) | (0.66) | (0.09) | (0.07) | (0.52) | (0.45) | (4.95) | (4.29) |

**Table S5.** The number of SNPs, non-synonymous SNPs, SVs, and indels with corresponding genes

| **Variety** | **Genes with**  **Non-synonymous**  **SNPs:SNPs** | **Genes with（>=10）**  **Non-synonymous SNPs:SNPs** | **Genes with**  **Frame-shift**  **InDels:InDel** | **Genes with**  **SV:SV** |
| --- | --- | --- | --- | --- |
| **‘Nagafu No.2’** | 23,111 : 171,520 | 5,734 : 107,364 | 1,681 : 1,834 | 3,431 : 3,963 |
| **‘Qingaun’** | 21,400 : 147,090 | 4,693 : 87,762 | 1,345 : 1,451 | 2,815 : 3,196 |

**Table S6.** The *cis*-elements in *FT* gene promoter sequences between ‘Nagafu No. 2’ and ‘Qinguan’ apple varieties.

| ***cis*-element** | **Function** | **Number of *cis*-element** | |
| --- | --- | --- | --- |
|  |  | **‘Nagafu No. 2’** | **‘Qinguan’** |
| AAGAA-motif | DNAse I footprinting | 2 | 2 |
| ABRE | cis-acting element involved in the abscisic acid responsiveness | 1 | 1 |
| AE-box | part of a module for light response | 1 | 1 |
| ARE | cis-acting regulatory element essential for the anaerobic induction | 2 | 2 |
| Box 4 | part of a conserved DNA module involved in light responsiveness | 2 | 2 |
| Box II | part of a light responsive element | 1 | 1 |
| CAAT- box | common cis-acting element in promoter and enhancer regions | 46 | 46 |
| CGTCA-motif | cis-acting regulatory element involved in the MeJA-responsiveness | 1 | 1 |
| G-Box | cis-acting regulatory element involved in light responsiveness | 1 | 1 |
| G-box | cis-acting regulatory element involved in light responsiveness | 1 | 1 |
| GAG-motif | part of a light responsive element | 1 | 1 |
| GARE-motif | gibberellin-responsive element | 1 | 1 |
| GATA-motif | part of a light responsive element | 1 | 1 |
| GC-motif | enhancer-like element involved in anoxic specific inducibility | 1 | 1 |
| I-box | part of a light responsive element | 1 | 1 |
| MBS | MYB Binding Site | 2 | 2 |
| MNF1 | light responsive element | 1 | 1 |
| MRE | MYB binding site involved in light responsiveness | 1 | 1 |
| O2-site | cis-acting regulatory element involved in zein metabolism regulation | 2 | 2 |
| Skn-1_motif | cis-acting regulatory element required for endosperm expression | 2 | 2 |
| Sp1 | light responsive element | 3 | 3 |
| TATA-box | core promoter element around -30 of transcription start | 56 | 56 |
| TC-rich repeats | cis-acting element involved in defense and stress responsiveness | 1 | 1 |
| TCCC-motif | part of a light responsive element | 1 | 1 |
| TCT-motif | part of a light responsive element | 2 | 2 |
| TGACG-motif | cis-acting regulatory element involved in the MeJA-responsiveness | 1 | 1 |
| Circadian | cis-acting regulatory element involved in circadian control | 4 | 4 |
| AAGAA-motif | DNAse I footprinting | 2 | 2 |
| ABRE | cis-acting element involved in the abscisic acid responsiveness | 1 | 1 |
| AE-box | part of a module for light response | 1 | 1 |
| ARE | cis-acting regulatory element essential for the anaerobic induction | 2 | 2 |
| Box 4 | part of a conserved DNA module involved in light responsiveness | 2 | 2 |
| Box II | part of a light responsive element | 1 | 1 |

**Fig.S1**

**
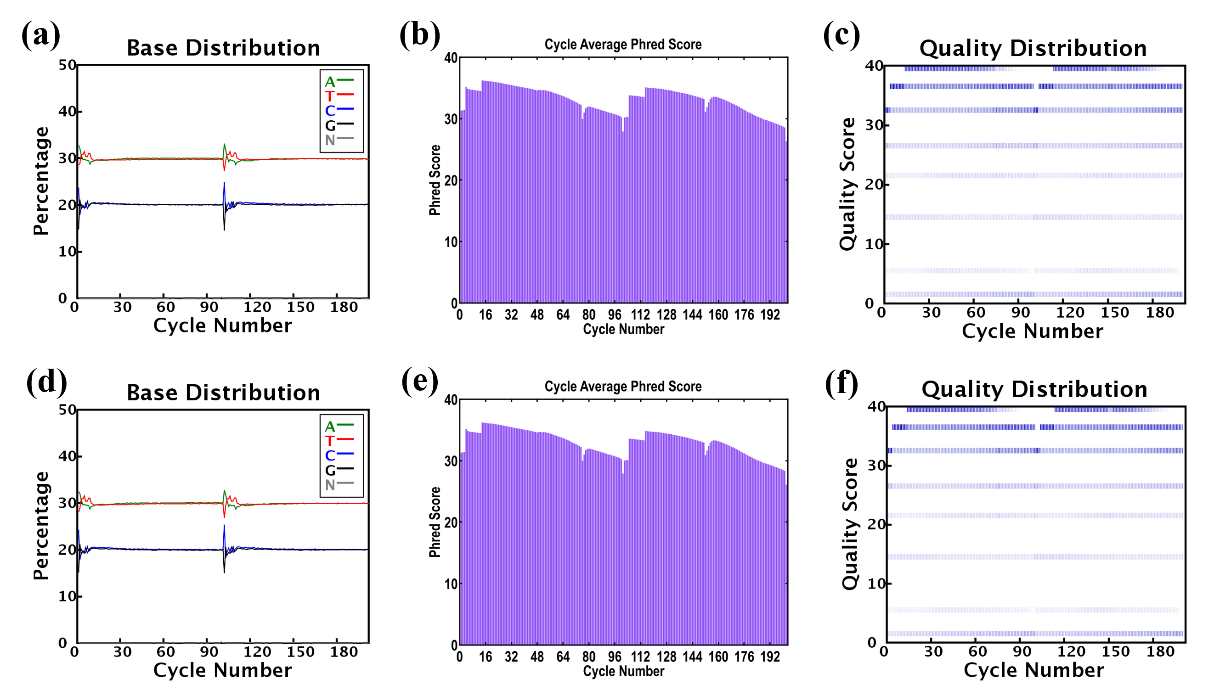
**

**Fig.S2**

**
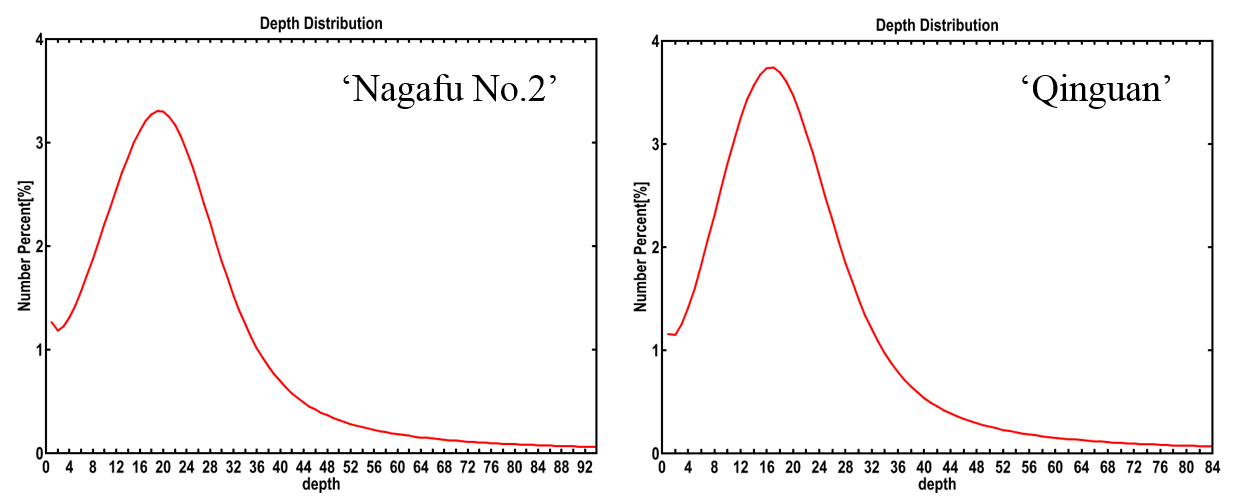
**

**Fig.S3**

**
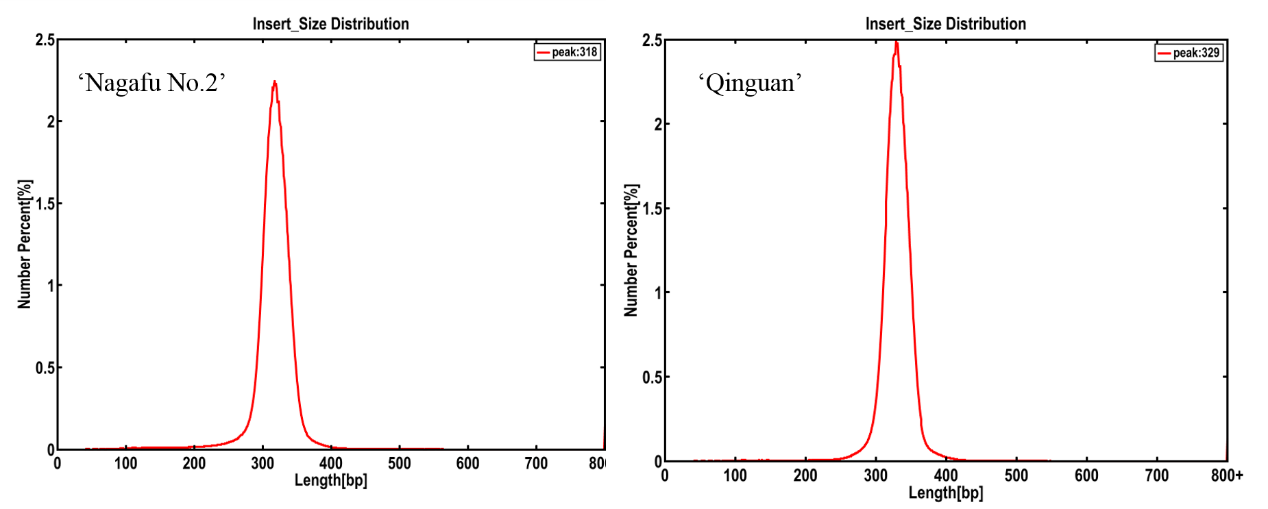
**

**Fig.S4**

**
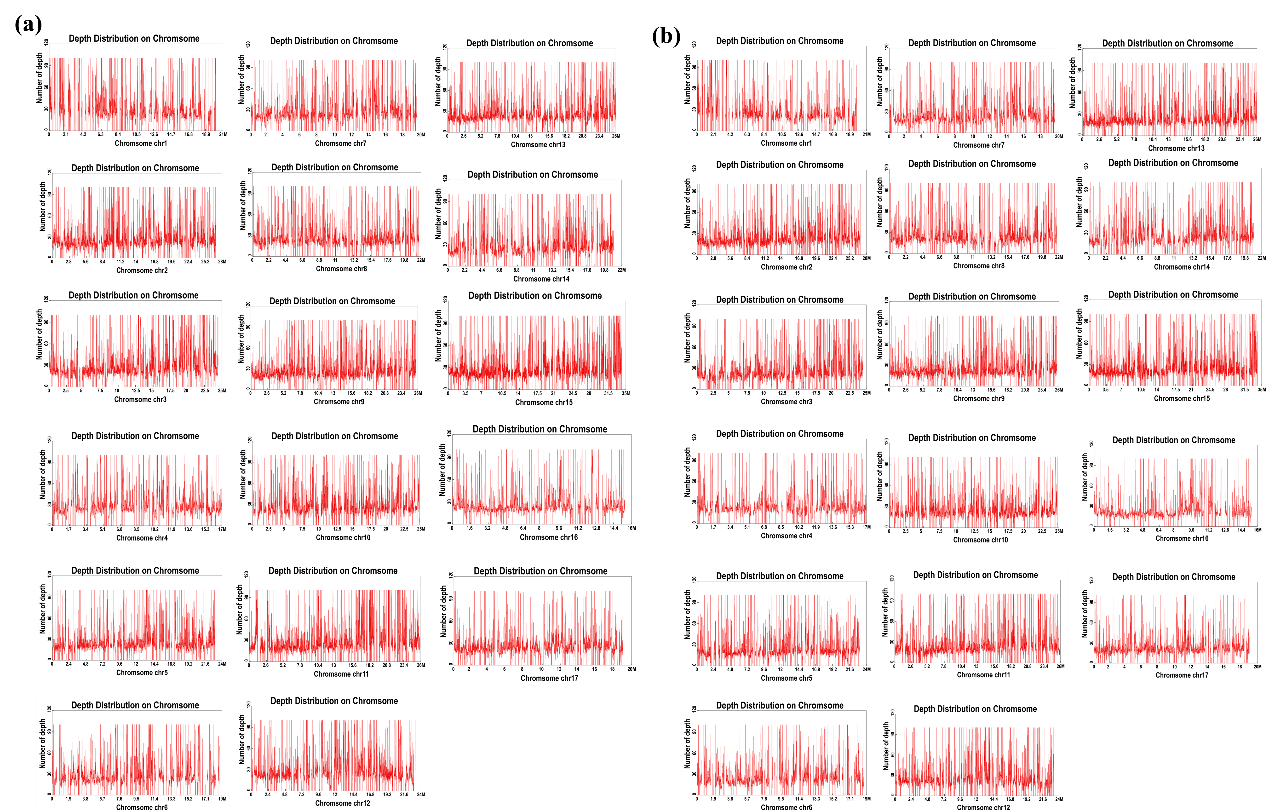
**

**Fig.S5**

**
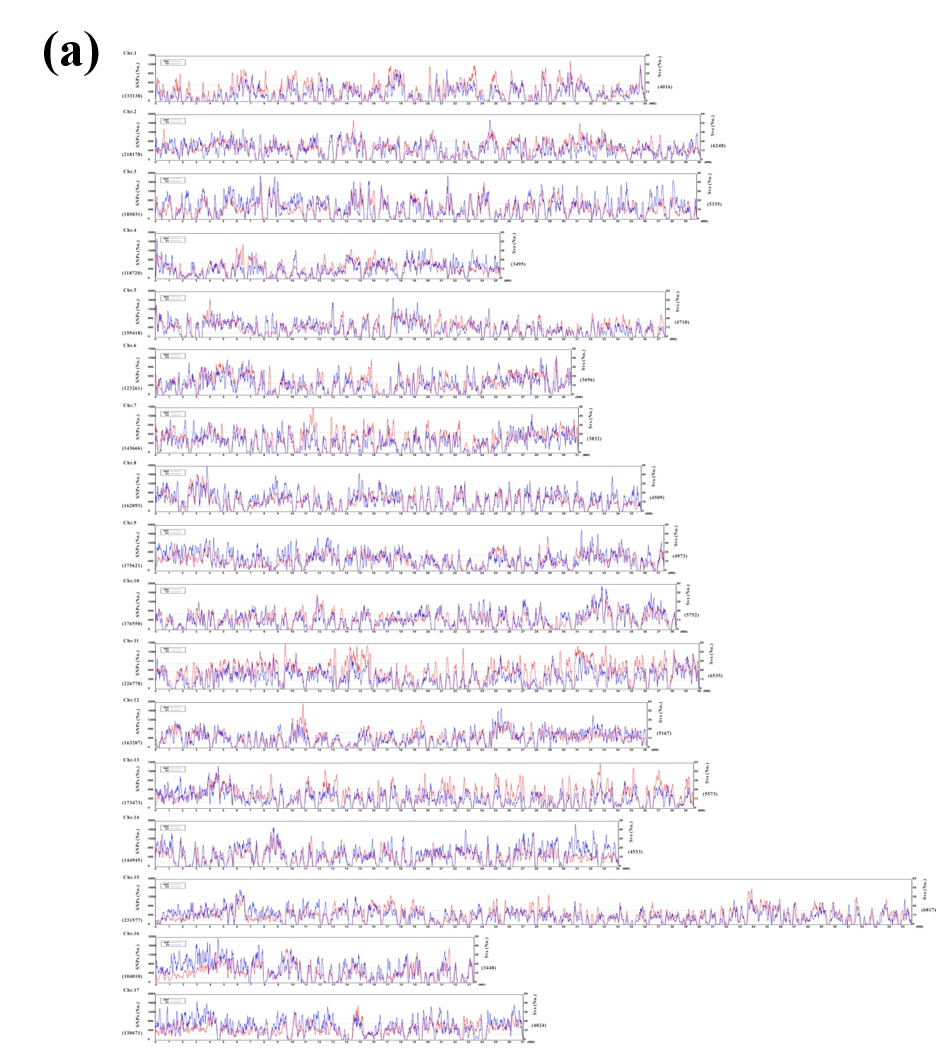
**

**
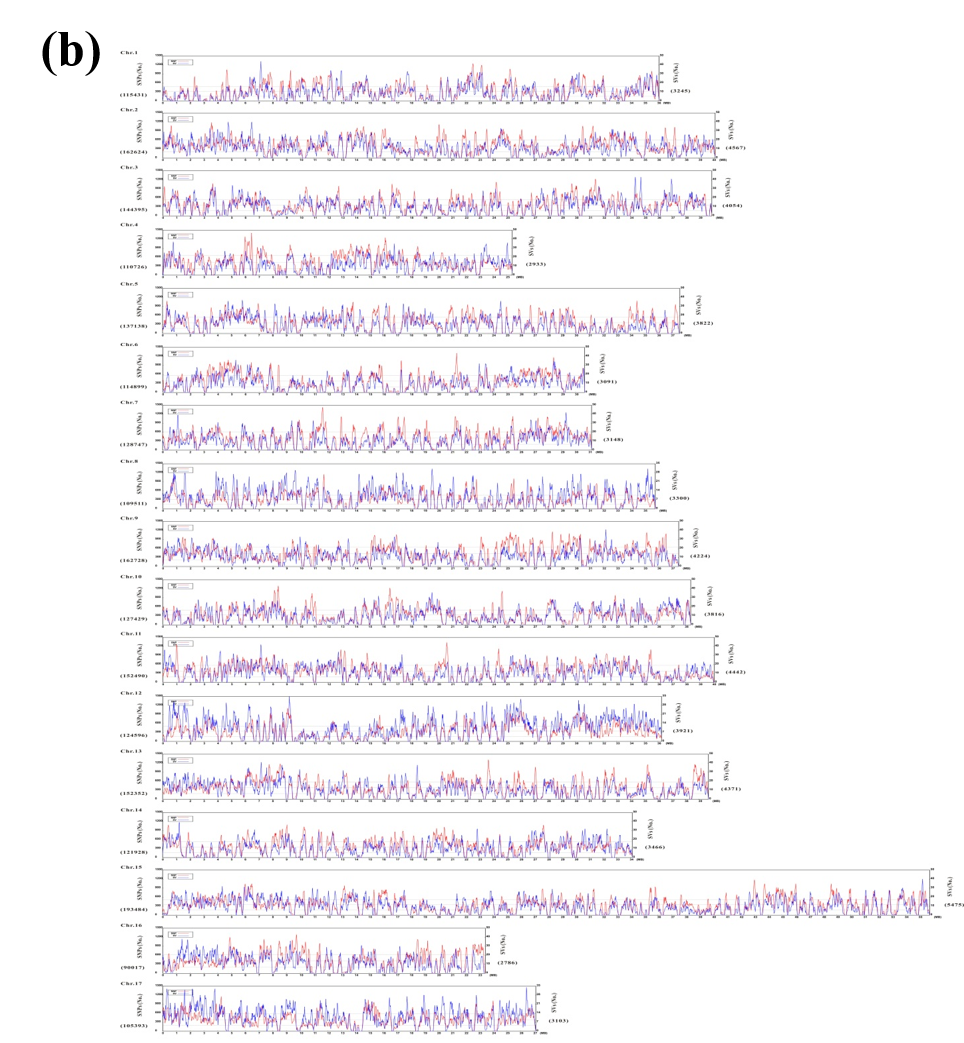
**

**Fig.S6**

**
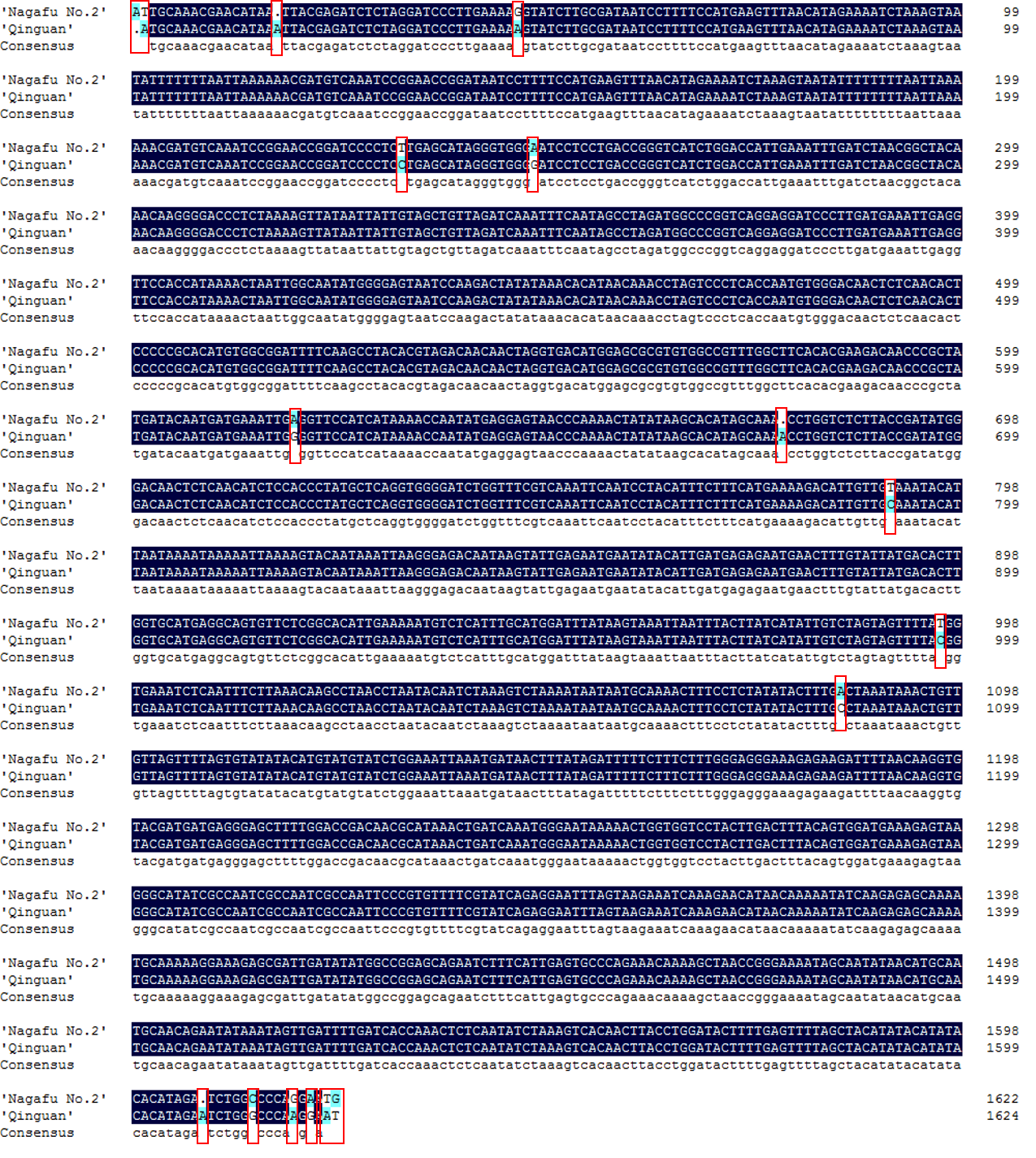
**
